# Impact of COVID-19 Vaccination in Thailand: Averted Deaths and Severe Infections Across Age Groups

**DOI:** 10.3390/tropicalmed9120286

**Published:** 2024-11-22

**Authors:** Chaiwat Wilasang, Pikkanet Suttirat, Dhammika Leshan Wannigama, Mohan Amarasiri, Sudarat Chadsuthi, Charin Modchang

**Affiliations:** 1 Biophysics Group, Department of Physics, Faculty of Science, Mahidol University, Bangkok 10400, Thailand; chaiwat.wilas@kmutt.ac.th (C.W.); pikkanet.sui@student.mahidol.edu (P.S.); 2 Center for Disease Modeling, Faculty of Science, Mahidol University, Bangkok 10400, Thailand; 3 Department of Infectious Diseases and Infection Control, Yamagata Prefectural Central Hospital, Yamagata 990-2292, Japan; dhammika.l@chula.ac.th; 4 Department of Microbiology, Faculty of Medicine, Chulalongkorn University, King Chulalongkorn Memorial Hospital, Thai Red Cross Society, Bangkok 10400, Thailand; 5 Center of Excellence in Antimicrobial Resistance and Stewardship, Faculty of Medicine, Chulalongkorn University, Bangkok 10400, Thailand; 6 School of Medicine, Faculty of Health and Medical Sciences, The University of Western Australia, Nedlands, Western Australia, Perth, WA 6009, Australia; 7 Biofilms and Antimicrobial Resistance Consortium of ODA Receiving Countries, The University of Sheffield, Sheffield S10 2TN, UK; 8 Pathogen Hunter’s Research Collaborative Team, Department of Infectious Diseases and Infection Control, Yamagata Prefectural Central Hospital, Yamagata 990-2292, Japan; 9 Environmental Water Quality Engineering Laboratory, Department of Civil and Environmental Engineer-ing, Graduate School of Engineering, Tohoku University, 6-6-06, Aramaki, Aoba-ku, Sendai 980-8579, Japan; mohan.amarasiri.b5@tohoku.ac.jp; 10 Department of Physics, Faculty of Science, Naresuan University, Phitsanulok 65000, Thailand; 11 Centre of Excellence in Mathematics, Ministry of Higher Education, Science, Research and Innovation, Bangkok 10400, Thailand; 12 Thailand Center of Excellence in Physics, Ministry of Higher Education, Science, Research and Innovation, 328 Si Ayutthaya Road, Bangkok 10400, Thailand

**Keywords:** COVID-19, vaccination, mathematical modeling, averted deaths, Thailand

## Abstract

The COVID-19 pandemic has underscored the pivotal role of vaccines in mitigating the devastating impact of the virus. In Thailand, the vaccination campaign against SARS-CoV-2 began on 28 February 2021, initially prioritizing healthcare professionals before expanding into a nationwide effort on 7 June 2021. This study employs a mathematical model of COVID-19 transmission with vaccination to analyze the impact of Thailand’s COVID-19 vaccination program from 1 March 2021 to 31 December 2022. We specifically assess the potential loss of lives and occurrence of severe infections across various age groups in a hypothetical scenario where vaccines were not administered. By fitting our model with officially reported COVID-19 death data, our analysis reveals that vaccination efforts prevented a total of 300,234 deaths (95% confidence interval: 295,938–304,349) and averted 1.60 million severe COVID-19 infections (95% confidence interval: 1.54–1.65 million). Notably, the elderly population over 80 years old benefited the most from vaccination, with an estimated 84,518 lives saved, constituting 4.28% of this age group. Furthermore, individuals aged between 70 and 74 years experienced the highest reduction in severe infections, with vaccination potentially preventing 8.35% of this age bracket from developing severe COVID-19.

## 1. Introduction

The COVID-19 pandemic, caused by severe acute respiratory syndrome coronavirus 2 (SARS-CoV-2), has presented unprecedented challenges to global public health since its emergence in Wuhan, China, in late 2019 [1,2]. As the virus rapidly spread across nations in early 2020, governments worldwide implemented a range of non-pharmaceutical interventions (NPIs) to mitigate transmission. These measures, spanning from localized physical distancing to widespread lockdowns, played a crucial role in curbing the virus’s spread and alleviating pressure on healthcare systems [3,4,5]. However, while effective in disease containment, these restrictions came at considerable costs to personal freedoms and had substantial impacts on people’s financial, psychological, and emotional well-being [6,7].

In this context, the development and deployment of vaccines emerged as a pivotal tool in epidemic control [8,9]. The global COVID-19 vaccine rollout commenced in December 2020, initially concentrated in high-income countries [10,11,12]. Early successes of COVID-19 vaccines became evident in countries like Israel and the United Kingdom, where significant reductions in cases, hospitalizations, and fatalities were observed [13,14,15,16]. This prompted a push for global vaccination efforts. However, it is important to note that the early evaluation of vaccination programs primarily occurred in Western countries with high incidence rates, contrasting with several Western Pacific nations, including Thailand, which maintained lower levels of epidemic activity during the initial stages of the pandemic [11,17].

Thailand’s experience with COVID-19 has been unique and evolving. The country reported the first COVID-19 case outside mainland China in early 2020 [17]. Initially, Thailand effectively managed and controlled the first two waves of the epidemic, resulting in a relatively low cumulative total of 29,127 cases and 95 deaths as of 29 March 2021 [11]. However, the situation changed dramatically in early April 2021 with the onset of a third transmission wave, driven by the Alpha variant, followed by a fourth wave in late May 2021, caused by the Delta variant. These imported variants triggered Thailand’s most significant COVID-19 outbreak since the pandemic’s onset [11,18,19].

In response to this surge, the Thai government imposed stringent lockdown measures from 20 July to 1 November 2021 [11,20]. Concurrently, Thailand’s vaccination efforts were ramping up. The initial doses of the CoronaVac vaccine (CV) and the ChAdOx1 nCoV-19 vaccine (AZ) were administered to healthcare workers on 28 February and 16 March 2021, respectively [11]. The nationwide vaccination campaign officially launched on 7 June 2021. Faced with the emergence of the Delta variant and limited AZ vaccine availability, the Department of Disease Control implemented various strategies, including a heterologous vaccination approach combining CV and AZ vaccines [11].

Evaluation of vaccination programs typically involves two main aspects: individual and population levels. While individual-level assessments comparing vaccinated and unvaccinated persons are well-documented across different countries, including Thailand [16,21,22], a comprehensive evaluation of vaccination strategies necessitates population-level analyses. These analyses consider factors such as decreased infection opportunities, lower transmission rates, and the indirect protective effects of vaccination, including herd immunity [23,24]. Population-level analyses provide a holistic view of the impact of vaccination programs on the overall health of the community.

This study aimed to quantify the impact of COVID-19 vaccination and estimate the number of lives saved from COVID-19 in Thailand. Through simulations of counterfactual scenarios, we determine the overall effectiveness of COVID-19 vaccination in Thailand up to 31 December 2022, three months after the Center for COVID-19 Situation Administration (CCSA) declared the end of the nationwide COVID-19 emergency. By comparing the actual outcomes with simulated scenarios in which vaccination was not implemented, we can assess the extent to which vaccination has reduced COVID-19 morbidity and mortality in Thailand. Our findings would provide important insights into the effectiveness of Thailand’s vaccination program and the role of vaccines in mitigating the impact of the pandemic, as well as valuable information for future public health strategies and vaccine deployment efforts in Thailand and other countries facing similar challenges.

## 2. Materials and Methods

### 2.1. Data Sources

Our study relied on comprehensive datasets from authoritative sources to ensure robust analysis. We obtained daily confirmed COVID-19 deaths and vaccine doses administered in Thailand from the Department of Disease Control (DDC) of the Ministry of Public Health, Thailand. The DDC is the authoritative government agency responsible for collecting and maintaining this information. While the original dataset is not publicly available for direct download, we have included the anonymized data used in this study as Supplementary Microsoft Excel Files to enable other researchers to access the data. To construct our baseline model for COVID-19 transmission incorporating vaccination, we employed mortality data from 1 March 2020 to 31 December 2022. This extensive timeframe allowed us to capture the full trajectory of the pandemic in Thailand, including pre-vaccination periods and subsequent vaccine rollout phases. For demographic context, we also referenced the 2020 World Population Prospects report, which estimated Thailand’s population at around 71 million [25]. These demographic data were instrumental in stratifying our analyses by age groups, enabling a more nuanced understanding of COVID-19’s impact and vaccination efficacy across different segments of the Thai population.

### 2.2. Vaccination Rollout Dynamics

We obtained detailed vaccine rollout and coverage data from the Department of Disease Control of Thailand, focusing on the period from 1 March 2021 to 31 December 2022. This 22-month timeframe captures several key phases of Thailand’s vaccination efforts: (1) initial rollout to healthcare workers (February–March 2021), (2) nationwide campaign launch (7 June 2021), (3) accelerated vaccination during the Delta variant surge (mid-2021), and (4) administration of booster doses and adaptation to new variants (late 2021–2022). Thailand began administering COVID-19 vaccines to healthcare workers on 28 February 2021. However, the nationwide vaccination rollout did not commence until 7 June 2021. Initially, vaccine shortages compelled the country to adopt various homologous and heterologous vaccination approaches. For a comprehensive overview of the dynamics and evolution of Thailand’s vaccination campaign, refer to [11,26].

### 2.3. Transmission Model

To estimate the impact of COVID-19 vaccination in Thailand, we employed a previously published deterministic compartmental epidemiological model specifically designed to simulate the transmission dynamics of COVID-19 [23,27,28]. This age-structured SEIR (Susceptible, Exposed, Infectious, Recovered) model categorizes individuals into compartments and tracks transitions between these states over time using systems of ordinary differential equations (see Figures S1 and S2 in the Supplementary Materials for model details). Our model extends the basic SEIR framework to include additional compartments based on specific characteristics of the disease, population, and interventions such as vaccination impacts. The diagram in the Supplementary Materials represents state transitions between epidemiological compartments, not causal relationships.

The model was implemented in the R programming language using the squire package (https://mrc-ide.github.io/squire/ (accessed on 2 August 2023)) as the primary package, with additional packages such as stats for optimization and ggplot2 for visualization.

We calibrated the model parameters to align with the best estimates of key factors driving the spread of SARS-CoV-2 in Thailand (Table 1). These included hospitalization and treatment-related parameters, such as the mean durations of hospitalization for severe and non-severe cases, as well as the number of available hospital beds and ICU beds in Thailand. To initialize the simulation, we uniformly distributed 21 cases across age groups on 1 March 2020, based on reported data. This initialization date corresponds to the early stages of the COVID-19 outbreak in Thailand, allowing the model to capture the full trajectory of the epidemic in the country.

The complete set of model equations and implementation details can be found in the section on mathematical model equations in the Supplementary Materials.

### 2.4. Transmission Model Fitting

To assess the impact of Thailand’s COVID-19 vaccination program, we fitted the model to confirmed reported deaths as a baseline scenario. The central aspect of the calibration procedure was estimating the time-varying reproduction number, *R_t_*, which serves as a dynamic indicator of COVID-19 transmission dynamics. This parameter allowed us to incorporate the potential impact of various factors, including vaccination and other non-pharmaceutical interventions, during the pandemic. We adjusted *R_t_* every 14 days to account for potential shifts in epidemiological patterns.

We employed a Maximum Likelihood Estimation (MLE) approach, which does not assume data normality, to identify *R_t_* that maximizes the likelihood of observing the reported data under our model framework [31]. The best fit was determined by maximum log-likelihood using the “logLik” function in R, where we assumed that the errors between observed and predicted values follow a normal distribution due to random noise in constructing the likelihood function [32,33,34,35]. Following Cole et al. (2014), MLE provides robust parameter estimates that maximize the probability of observed outcomes, even when the data may not conform to normality [31]. We also utilized the Likelihood Ratio Test (LRT) to compute the 95% partial likelihood-based confidence interval for the fitted parameters [36], establishing upper and lower bounds for *R_t_* (see Table S1 in the Supplementary Materials).

### 2.5. Quantifying the Impact of Vaccination on Averting COVID-19 Mortality and Severe Infection

To isolate the specific effects of vaccination and highlight its crucial role in mitigating severe outcomes, we simulated a hypothetical scenario in which vaccines were unavailable. In this counterfactual scenario, the vaccination rate was set to zero, while the estimated time-varying reproduction number (*R_t_*) remained consistent with the baseline model. By comparing the COVID-19 mortality and severe cases in the baseline scenario to those in the no-vaccination scenario, we quantified the impact of vaccination in terms of prevented deaths and averted severe cases. Here, COVID-19 mortality is defined as all deaths directly resulting from COVID-19 infection. This approach allowed us to evaluate the effectiveness of vaccination as an intervention measure, independent of other factors influencing the pandemic’s trajectory.

## 3. Results

### 3.1. Baseline Model Fitting and Counterfactual Scenario

**Figure 1:**
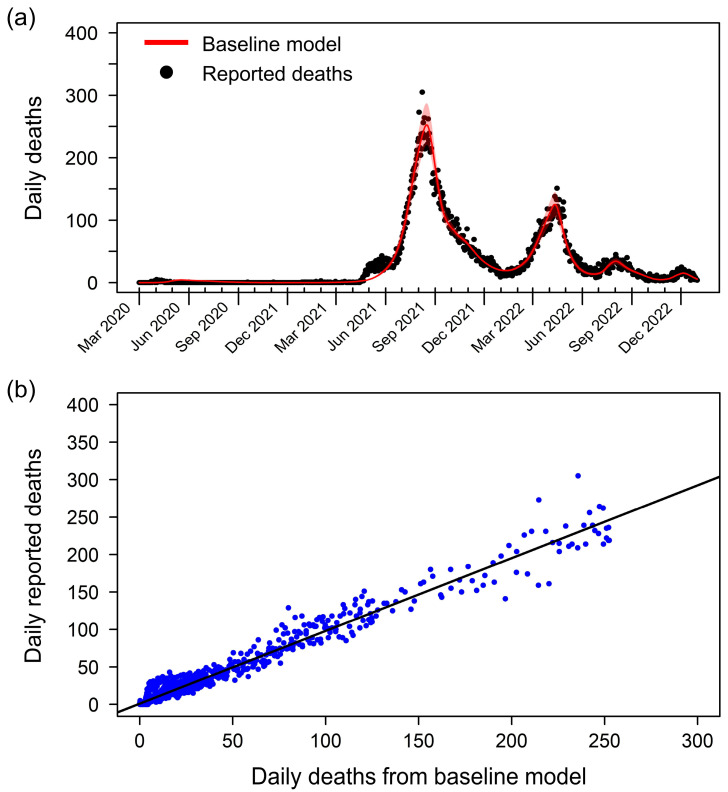
Baseline model fitting and validation of the model’s accuracy. (**a**) Comparison of reported daily deaths (black dots) and daily death counts generated by the baseline model (red line). The baseline model effectively captures the trends and peaks in COVID-19 mortality in Thailand. The shaded area represents the 95% partial likelihood-based confidence interval, indicating the uncertainty in the model estimates. (**b**) Scatter plot comparing the estimated deaths from the baseline model (x-axis) and the reported deaths (y-axis). Each blue dot represents a data point from the study period. The black line shows the linear regression between the estimated and reported deaths, with an R-square value of 0.9686.

Our fitted transmission model effectively captures the baseline dynamics of COVID-19 mortality in Thailand (Figure 1a). The model’s estimated cumulative death toll of 33,652 by 31 December 2022 closely matches the officially reported number of 33,708. Notably, the reported deaths initially peaked on 8 August 2021, reaching 305 deaths per day, followed by a second peak on 13 April 2022, with 221 deaths per day. The scatter plot comparing the baseline model and reported deaths reveals a strong correlation, evidenced by a high R-square value of 0.9686 (Figure 1b). Additional model performance metrics, including a root mean square error (RMSE) of 19.51 deaths per day and a mean absolute error (MAE) of 9.37 deaths per day, further underscore the model’s accuracy. These metrics indicate a robust fit, where the daily deaths predicted by the baseline model closely match the observed values, validating the model’s ability to capture the dynamics of COVID-19 mortality in Thailand accurately.

**Figure 2:**
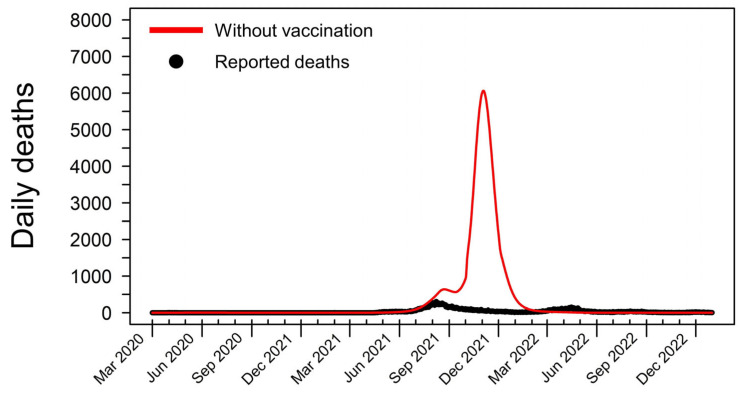
Simulated COVID-19 deaths in Thailand in a hypothetical scenario without vaccination. This graph shows the hypothetical daily death toll from 1 March 2020 to 31 December 2022, assuming no vaccines were administered throughout the pandemic. In this simulated scenario, the cumulative number of COVID-19 fatalities reaches 333,886, nearly ten times higher than the actual reported deaths during this period. The model predicts a dramatic surge in mortality beginning in early June 2021, coinciding with the emergence and rapid spread of the highly transmissible Delta variant in Thailand. Without the mitigating effects of vaccination, the daily death count peaks at 6061 on 3 November 2021, highlighting the crucial role of vaccines in preventing such catastrophic outcomes.

To assess the impact of vaccination on the pandemic’s trajectory, we simulated a counterfactual scenario in which no vaccination was implemented. Under this hypothetical scenario, the total number of deaths could have reached a staggering 333,886 by 31 December 2022, nearly ten times the actual reported deaths (Figure 2). The number of daily deaths in this scenario began to rise sharply in early June 2021, coinciding with the emergence of the Delta variant in Thailand. The peak of this simulated wave occurred on 3 November 2021, with 6061 deaths per day. This stark contrast between the baseline model and the counterfactual scenario underscores the critical role of vaccination in mitigating the loss of life during the COVID-19 pandemic in Thailand.

### 3.2. Vaccination and Averted Deaths in Thailand

**Figure 3:**
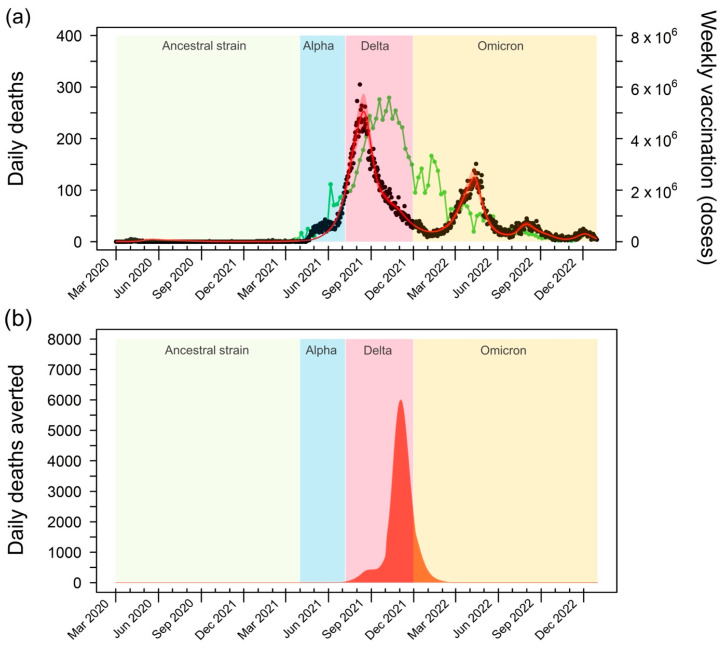
Impact of COVID-19 vaccination on mortality in Thailand. (**a**) The green line depicts the weekly vaccine doses administered in Thailand from 1 March 2021 to 31 December 2022. A substantial increase in vaccination rates is evident between July and November 2021, with a peak of 5.59 million doses per week in October 2021. This surge in vaccination coincides with a decline in reported daily deaths (black dots), highlighting the effectiveness of the vaccination campaign in reducing COVID-19 mortality. (**b**) Averted deaths due to vaccination, calculated as the difference between the projected deaths in the baseline model and a hypothetical scenario without vaccination. The graph illustrates the substantial number of lives saved by the vaccination efforts, particularly during the peak of the Delta variant’s transmission. The shaded areas represent the periods of dominance for different SARS-CoV-2 variants in Thailand: the Ancestral strain (1 March 2020 to 31 March 2021), Alpha variant (1 April 2021 to 7 June 2021), Delta variant (8 June 2021 to 30 November 2021), and Omicron variant (1 December 2021 to 31 December 2022) [18].

The rollout of COVID-19 vaccines in Thailand faced initial challenges, with the vaccination rate lagging behind the surge in deaths driven by the highly transmissible Delta variant. However, as the vaccination campaign gained momentum and reached its peak, a significant reduction in fatalities was observed (Figure 3a). This decline in mortality, despite the continued spread of the Delta variant, underscores the effectiveness of the vaccination program in saving lives. The subsequent emergence of the Omicron variant led to a smaller secondary peak in mortality, highlighting the ongoing challenges posed by the evolving nature of the pandemic and the need for sustained vaccination efforts.

To quantify the impact of vaccination on averting deaths, we compared the estimated number of deaths in the baseline model, which accounts for the real-world vaccination program, with the counterfactual scenario in which no vaccines were administered (Figure 3b). This analysis reveals the substantial number of lives saved by the vaccination efforts, particularly during the peak of the Delta variant’s spread. Notably, the model estimates that vaccination averted up to 6061 deaths per day at the height of the Delta wave. This finding emphasizes the crucial role of vaccination in mitigating the most severe consequences of the pandemic, especially during periods of heightened transmission.

### 3.3. Prevented Deaths and Reduced Severity Across Age Groups

**Figure 4:**
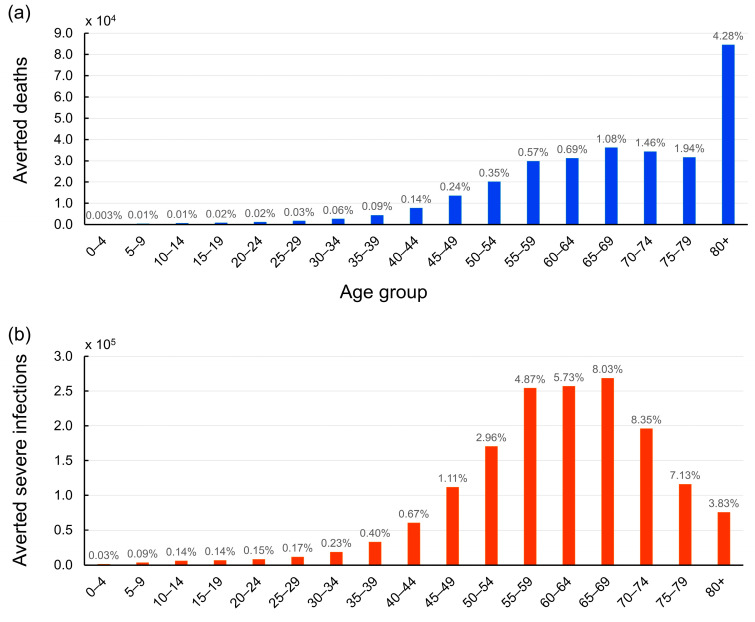
Age-stratified impact of COVID-19 vaccination on averted deaths and severe cases in Thailand. (**a**) The bar graph illustrates the number of lives saved across different age groups due to vaccination efforts from 1 March 2021 to 31 December 2022. The number above each bar represents the proportion of lives saved relative to the total population within each age group. The elderly population, particularly those over 80 years old, benefited the most from vaccination, with 84,518 deaths averted, constituting 4.28% of this age group. (**b**) The bar graph depicts the number of severe COVID-19 cases prevented by vaccination across various age groups. The number above each bar indicates the percentage of cases that were prevented from developing severe infections relative to the total population in each age group. Individuals aged between 70 and 74 years experienced the highest reduction in severe cases, with vaccination potentially preventing 8.35% of this age bracket from severe COVID-19.

Figure 4 illustrates the impact of vaccination on averting deaths and reducing severe cases across different age groups. Our analysis reveals a clear trend: the protective effect of vaccination against COVID-19 mortality increases significantly with age. In particular, vaccination has played a crucial role in preventing 84,518 fatalities among individuals over 80 years old, equivalent to saving the lives of 4.28% of this age group (Figure 4a).

Interestingly, when comparing the mitigation of severe cases to the prevention of deaths, our analysis uncovers a distinct pattern. The age group benefiting most from vaccination in terms of reduced disease severity upon infection is individuals aged between 70 and 74 years. The data suggest that vaccination has potentially prevented 8.35% of this age group from developing severe COVID-19 (Figure 4b). This finding indicates the importance of vaccines not only in preventing deaths but also in reducing the burden of severe illness on healthcare systems. By lowering the incidence of severe cases, vaccination helps to alleviate the strain on hospitals and intensive care units, ensuring better care for those who require hospitalization.

Overall, our findings demonstrate the substantial impact of Thailand’s vaccination efforts in mitigating the pandemic’s toll on public health. The vaccination campaign has averted an estimated 1.60 million severe COVID-19 infections (95% confidence interval: 1.54 to 1.65 million) across all age groups. This significant reduction in severe cases emphasizes the broad-reaching benefits of vaccination in protecting individuals from the most serious consequences of COVID-19 infection.

## 4. Discussion

Our study presents a comprehensive assessment of the impact of Thailand’s COVID-19 vaccination program from its initiation in February 2021 to the end of December 2022. By employing a mathematical model to simulate the transmission dynamics of COVID-19 and the effects of vaccination, we have quantified the pivotal role that vaccination has played in mitigating the pandemic’s impact in the country. Our analysis reveals that vaccination efforts have prevented an estimated 300,234 deaths and averted approximately 1.60 million severe COVID-19 infections nationwide. These findings underscore the critical importance of vaccination as a key public health measure in combating the COVID-19 pandemic, highlighting the need for continued efforts to expand vaccine coverage and protect populations from the virus.

Evaluating the direct and indirect effects of vaccination programs on COVID-19 mortality presents challenges due to the inability to observe a counterfactual scenario in which vaccinations were not administered. Mathematical models, such as the one employed in our study, serve as valuable tools for assessing the impact of vaccination campaigns on epidemic dynamics [23,24]. By simulating a hypothetical scenario without vaccinations, our analysis reveals the potential devastation that COVID-19 could have inflicted, claiming up to 333,886 lives in Thailand by the end of December 2022. This stark contrast highlights the remarkable effectiveness of COVID-19 vaccines in significantly reducing mortality rates, even in the face of highly transmissible variants such as Delta and Omicron [11,37]. Moreover, our analysis sheds light on the demographic distribution of vaccination benefits, revealing that the elderly population, particularly those over 80 years old, received the most substantial protection against death. This finding aligns with the global understanding that older age groups are at a higher risk of severe outcomes from COVID-19 infection [38]. Consequently, the targeted vaccination of older individuals has emerged as a critical strategy in reducing overall mortality rates [11]. In contrast, we find that the population aged 70 to 74 years saw the most significant decrease in severe infections. This might be because individuals aged 70 to 74 years have a high proportion of infections requiring hospitalization and ICU admission, but their infection fatality ratio (IFR) is relatively lower compared to those aged 80 years and above [27]. Consequently, the 70 to 74 age group experienced a more substantial reduction in severe infections and ICU requirements, rather than mortality, following vaccination.

Our study builds upon previous work by Watson et al. [23], which refined earlier models by Hogan et al. [27] and Walker et al. [28] to quantify the global impact of COVID-19 vaccination programs. While these studies provided valuable insights, they did not specifically investigate the impact of vaccination in Thailand. We adapted the model proposed by Watson et al. [23] to evaluate the effectiveness of COVID-19 vaccination in the Thai context, incorporating country-specific parameters such as hospital and ICU bed capacity to better describe the dynamics of COVID-19 mortality. Furthermore, we extended the study period to 31 December 2022, capturing a more comprehensive trajectory of the pandemic in Thailand, particularly considering the later start of the nationwide vaccination campaign compared to high-income countries. Our analysis relied on authoritative data sources, including daily confirmed COVID-19 deaths and vaccine doses administered, obtained from the Department of Disease Control of the Ministry of Public Health, Thailand. The epidemiological characteristics specific to Thailand and the extended study timeframe were crucial for providing a comprehensive assessment of the substantial impact of COVID-19 vaccination in the country, highlighting the lives saved and severe infections prevented during the first two years of the vaccination campaign.

While our study provides compelling evidence of the benefits of vaccination, it is crucial to acknowledge the limitations inherent in our approach. The mathematical model relies on several assumptions and estimates, including vaccine efficacy and the duration of immunity. These assumptions may introduce some uncertainty into our findings, and further research is needed to refine these estimates as more data become available. Additionally, our model did not explicitly account for the impact of non-pharmaceutical interventions, such as lockdowns and social distancing measures, which were implemented concurrently with the vaccination program. Instead, the effects of these interventions were implicitly included in the transmission dynamics through the estimation of the time-varying reproduction number, *R_t_*. Future research could aim to disentangle the effects of vaccination from other interventions to provide a clearer picture of the relative contributions of different strategies in controlling the pandemic. This would enable policymakers to make more informed decisions about the most effective measures to implement in response to future outbreaks. Moreover, incorporating data on vaccine hesitancy and inequitable access to vaccines could provide additional insights into the factors influencing the overall effectiveness of the vaccination program.

More broadly, our estimates of the impact of COVID-19 vaccination in Thailand should be considered in light of the considerable uncertainty inherent in such modeling efforts. Uncertainties in the true extent of the pandemic’s death toll, the epidemiological characteristics of circulating SARS-CoV-2 variants, and the specific vaccines administered in the country may influence the precision of our estimates. However, despite these limitations, our analysis provides a comprehensive assessment of the substantial impact of COVID-19 vaccination in Thailand, highlighting the hundreds of thousands of lives saved and millions of severe infections prevented during the first two years of the vaccination campaign. These results underscore the critical role of vaccines in mitigating the devastating effects of the pandemic and emphasize the importance of ongoing efforts to expand vaccine coverage and equity. Our findings also have implications for future vaccination strategies and pandemic preparedness in Thailand and other similar settings, highlighting the need for sustained investment in public health infrastructure and proactive measures to address potential challenges, such as vaccine hesitancy and equitable distribution.

## 5. Conclusions

In conclusion, this study provides compelling evidence of the substantial impact of Thailand’s COVID-19 vaccination program in preventing deaths and severe infections during the pandemic. Our mathematical modeling analysis reveals that vaccination efforts averted an estimated 300,234 deaths (95% confidence interval: 295,938–304,349) and prevented approximately 1.60 million severe COVID-19 infections (95% confidence interval: 1.54–1.65 million) between March 2021 and December 2022. The benefits of vaccination were particularly pronounced among elderly populations, with individuals over 80 years old experiencing the highest reduction in mortality (4.28% of age group) and those aged 70–74 years showing the greatest decrease in severe infections (8.35% of age group).

## Figures and Tables

**Table 1:** Model parameters and their values.

Parameters	Values	Descriptions/References
Time-varying reproduction number (*R_t_*)	See Table S1	
Transmission rate	-	Calculated from *R_t_*
Mean latent period	4.6 days	[27]
Mean duration of mild infection	2.1 days	[27]
Mean duration of severe infection prior to hospitalization	4.5 days	[27]
Mean duration of hospitalization for non-severe cases if survive	9.0 days	[27]
Mean duration of hospitalization for non-severe cases if die	9.0 days	[27]
Mean duration in ICU if survive	14.8 days	[27]
Mean duration in ICU if die	11.1 days	[27]
Mean duration in recovery after ICU	3.0 days	[27]
Mean vaccine efficacy against infection	60%	[27]
Mean vaccine efficacy against disease	70%	[27]
Mean duration of naturally acquired immunity	365 days	[27]
Vaccine duration of protection	446 days	Estimated from [23]
Probability of death if require critical care but do not receive it	90.5% (range 85%–95%)	[28]
Probability of death if require hospitalization but no hospital beds are available	60% (range 50–70%)	[28]
Number of hospital beds in Thailand	158,326	[29]
Number of ICU beds with ventilators in Thailand	13,184	[30]

## Data Availability

The authors confirm that the data supporting the findings of this study are available within the article and its Supplementary Materials.

## Supplementary Materials

The following supporting information can be downloaded at: https://www.mdpi.com/article/10.3390/tropicalmed9120286/s1, Figure S1: Schematic of the age-structure transmission model. The solid arrows represent transitions between epidemiological classes. The model compartments comprise nine epidemiological classes: susceptible (*S*), exposed (*E*), mild infections (*I*_Mild_), infections requiring hospitalization but not yet hospitalized (*I*_Case_), hospitalized infections requiring a general hospital bed (*I*_HOSP_), hospitalized infections requiring an ICU bed (*I*_ICU_), hospitalized infections stepping down from ICU and requiring a general hospital bed for recovery (*I*_Rec_), recovered (*R*), and dead (*D*); Figure S2: Schematic outlining the progression of hospitalized infections necessitating an ICU bed. Individuals are categorized based on their need for general hospital and ICU beds, respectively. Patients who secure a bed experience a reduced probability of mortality compared to those who do not. Specifically, weighted arrows depict the probabilities. The probability of death when hospitalization is required but no hospital beds are available is estimated at 60% (with a range of 50–70%). Similarly, the probability of death if critical care is required but not received is estimated at 90.5% (with a range of 85–95%); Table S1: Estimated biweekly time–varying reproduction number (*R_t_*), starting from 1 March 2020, to 31 December 2022; Table S2: Model parameters, values, and definitions used in the COVID–19 transmission model.
