# Supplementary material for: Impact of COVID-19 Vaccination in Thailand: Averted Deaths and Severe Infections Across Age Groups
Source: Trop Med Infect Dis. 2024 Nov 22;9(12):286. doi: 10.3390/tropicalmed9120286 (PMC11679040; doi:10.3390/tropicalmed9120286)
Supplement: Supplementary file 1 [file tropicalmed-09-00286-s001.zip › tropicalmed-3238058-supplementary_CM22Nov24.pdf]

Supplementary Materials:

Impact of COVID-19 Vaccination in Thailand: Averted Deaths and Severe Infections Across Age Groups

Chaiwat Wilasang [1,2], Pikkanet Suttirat [1,2], Dhammika Leshan Wannigama [3,4,5,6,7,8], Mohan Amarasiri [9], Sudarat Chadsuthi [10,*] and Charin Modchang [1,2,11,12,*]

[1] Biophysics Group, Department of Physics, Faculty of Science, Mahidol University, Bangkok 10400, Thailand; chaiwat.wilas@kmutt.ac.th (C.W.); pikkanet.sui@student.mahidol.edu (P.S.)
[2] Center for Disease Modeling, Faculty of Science, Mahidol University, Bangkok 10400, Thailand
[3] Department of Infectious Diseases and Infection Control, Yamagata Prefectural Central Hospital, Yamagata 990-2292, Japan; dhammika.l@chula.ac.th
[4] Department of Microbiology, Faculty of Medicine, Chulalongkorn University, King Chulalongkorn Memorial Hospital, Thai Red Cross Society, Bangkok 10400, Thailand
[5] Center of Excellence in Antimicrobial Resistance and Stewardship, Faculty of Medicine, Chulalongkorn University, Bangkok 10400, Thailand
[6] School of Medicine, Faculty of Health and Medical Sciences, The University of Western Australia, Nedlands, Western Australia, Perth, WA 6009, Australia
[7] Biofilms and Antimicrobial Resistance Consortium of ODA Receiving Countries, The University of Sheffield, Sheffield S10 2TN, UK
[8] Pathogen Hunter's Research Collaborative Team, Department of Infectious Diseases and Infection Control, Yamagata Prefectural Central Hospital, Yamagata 990-2292, Japan
[9] Environmental Water Quality Engineering Laboratory, Department of Civil and Environmental Engineer-ing, Graduate School of Engineering, Tohoku University, 6-6-06, Aramaki, Aoba-ku, Sendai 980-8579, Japan; mohan.amarasiri.b5@tohoku.ac.jp
[10] Department of Physics, Faculty of Science, Naresuan University, Phitsanulok 65000, Thailand
[11] Centre of Excellence in Mathematics, Ministry of Higher Education, Science, Research and Innovation, Bangkok 10400, Thailand
[12] Thailand Center of Excellence in Physics, Ministry of Higher Education, Science, Research and Innovation, 328 Si Ayutthaya Road, Bangkok 10400, Thailand
* Correspondence: sudaratc@nu.ac.th (S.C.); charin.mod@mahidol.edu (C.M.)

Model structure

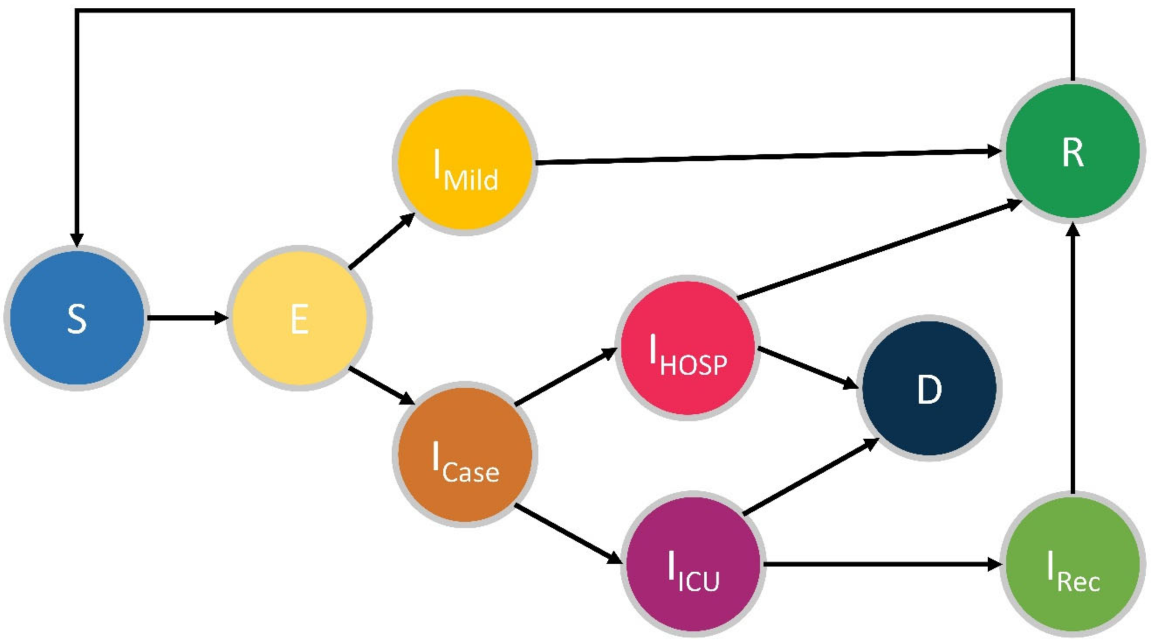

Figure S1. Schematic of the age-structure transmission model. The solid arrows represent transitions between epidemiological classes. The model compartments comprise nine epidemiological classes: susceptible (S), exposed (E), mild infections (I_{Mild}), infections requiring hospitalization but not yet hospitalized (I_{Case}), hospitalized infections requiring a general hospital bed (I_{HOSP}), hospitalized infections requiring an ICU bed (I_{ICU}), hospitalized infections stepping down from ICU and requiring a general hospital bed for recovery (I_{Rec}), recovered (R), and dead (D).

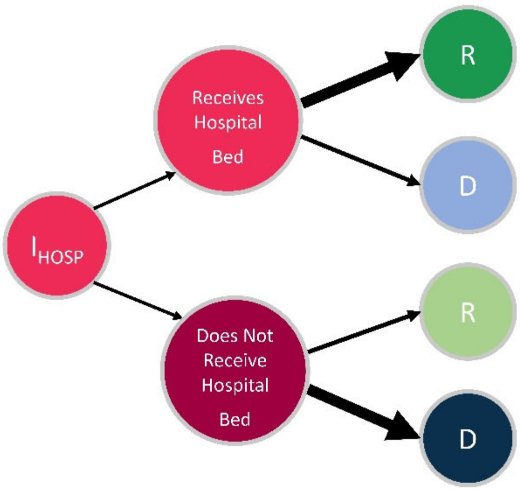

Figure S2. Schematic outlining the progression of hospitalized infections necessitating an ICU bed. Individuals are categorized based on their need for general hospital and ICU beds, respectively. Patients who secure a bed experience a reduced probability of mortality compared to those who do not. Specifically, weighted arrows depict the probabilities. The probability of death when hospitalization is required but no hospital beds are available is estimated at 60% (with a range of 50-70%). Similarly, the probability of death if critical care is required but not received is estimated at 90.5% (with a range of 85%-95%).

Mathematical model equations (the equations are based on [1])

1. Vaccination group v0 (unvaccinated)

$$\frac{dS(t,a,v_0)}{dt} = 2\rho R_2(t,a,v_0) - \beta\frac{S(t,a,v_0)}{N}\sum_{a'} c(a,a)\left[\sum_v\left(I_{MILD}(t,a',v) + I_{CASE}(t,a',v)\right)\right] - \kappa(a)S(t,a,v_0)$$

$$\frac{dE_1(t,a,v_0)}{dt} = \beta\frac{S(t,a,v_0)}{N}\sum_{a'} c(a,a)\left[\sum_v\left(I_{MILD}(t,a',v) + I_{CASE}(t,a',v)\right)\right] - 2\alpha E_1(t,a,v_0) - \kappa(a)E_1(t,a,v_0)$$

$$\frac{dE_2(t,a,v_0)}{dt} = 2\alpha E_1(t,a,v_0) - 2\alpha E_2(t,a,v_0) - \kappa(a)E_2(t,a,v_0)$$

$$\frac{dI_{MILD}(t,a,v_0)}{dt} = (1-\phi_1(a))\left(2\alpha E_2(t,a,v_0)\right) - \gamma_1 I_{MILD}(t,a,v_0)$$

$$\frac{dI_{CASE,0}(t,a,v_0)}{dt} = \phi_1(a)\left(2\alpha E_2(t,a,v_0)\right) - 2\gamma_2 I_{CASE,0}(t,a,v_0)$$

$$\frac{dI_{CASE,1}(t,a,v_0)}{dt} = 2\gamma_2 I_{CASE,0}(t,a,v_0) - 2\gamma_2 I_{CASE,1}(t,a,v_0)$$

$$\frac{dI_{HOSPITAL,0}(t,a,v_0,0,0)}{dt} = (1-\delta(H))\mu(a)(1-\phi_2(a))2\gamma_2 I_{CASE,1}(t,a,v_0) - 2\gamma_{3,0} I_{HOSPITAL,0}(t,a,v_0,0,0)$$

$$\frac{dI_{HOSPITAL,1}(t,a,v_0,0,0)}{dt} = 2\gamma_{3,0} I_{HOSPITAL,0}(t,a,v_0,0,0) - 2\gamma_{3,0} I_{HOSPITAL,1}(t,a,v_0,0,0)$$

$$\frac{dI_{HOSPITAL,0}(t,a,v_0,1,0)}{dt} = \delta(H)\mu(a)(1-\phi_2(a))2\gamma_2 I_{CASE,1}(t,a,v_0) - 2\gamma_{3,0} I_{HOSPITAL,0}(t,a,v_0,1,0)$$

$$\frac{dI_{HOSPITAL,1}(t,a,v_0,1,0)}{dt} = 2\gamma_{3,0} I_{HOSPITAL,0}(t,a,v_0,1,0) - 2\gamma_{3,0} I_{HOSPITAL,1}(t,a,v_0,1,0)$$

$$\frac{dI_{HOSPITAL,0}(t,a,v_0,0,1)}{dt} = (1-\delta(H))(1-\mu(a))(1-\phi_2(a))2\gamma_2 I_{CASE,1}(t,a,v_0) - 2\gamma_{3,1} I_{HOSPITAL,0}(t,a,v_0,0,1)$$

$$\frac{dI_{HOSPITAL,\,1}(t,a,v_0,0,1)}{dt} = 2\gamma_{3,1} I_{HOSPITAL,}(t,a,v_0,0,1) - 2\gamma_{3,1} I_{HOSPITAL,1}(t,a,v_0,0,1)$$

$$\frac{dI_{HOSPITAL,\,0}(t,a,v_0,1,1)}{dt} = \delta(H)(1-\mu(a))(1-\phi_2(a))2\gamma_2 I_{CASE,1}(t,a,v_0) - 2\gamma_{3,1} I_{HOSPITAL,0}(t,a,v_0,1,1)$$

$$\frac{dI_{HOSPITAL,\,1}(t,a,v_0,1,1)}{dt} = 2\gamma_{3,1} I_{HOSPITAL,}(t,a,v_0,1,1) - 2\gamma_{3,1} I_{HOSPITAL,1}(t,a,v_0,1,1)$$

$$\frac{dI_{ICU,0}(t,a,v_0,0,0)}{dt} = (1-\delta(ICU))\mu(a)\phi_2(a)2\gamma_2 I_{CASE,1}(t,a,v_0) - 2\gamma_{4,0} I_{ICU,0}(t,a,v_0,0,0)$$

$$\frac{dI_{ICU,1}(t,a,v_0,0,0)}{dt} = 2\gamma_{4,0} I_{ICU,0}(t,a,v_0,0,0) - 2\gamma_{4,0} I_{ICU,1}(t,a,v_0,0,0)$$

$$\frac{dI_{ICU,0}(t,a,v_0,1,0)}{dt} = \delta(ICU)\mu(a)\phi_2(a)2\gamma_2 I_{CASE,1}(t,a,v_0) - 2\gamma_{4,0} I_{ICU,0}(t,a,v_0,1,0)$$

$$\frac{dI_{CU,1}(t,a,v_0,1,0)}{dt} = 2\gamma_{4,0} I_{ICU,0}(t,a,v_0,1,0) - 2\gamma_{4,0} I_{ICU,1}(t,a,v_0,1,0)$$

$$\frac{dI_{ICU,0}(t,a,v_0,0,1)}{dt} = (1-\delta(ICU))(1-\mu(a))\phi_2(a)2\gamma_2 I_{CASE,1}(t,v_0,a) - 2\gamma_{4,1} I_{ICU,0}(t,a,v_0,0,1)$$

$$\frac{dI_{CU,1}(t,a,v_0,0,1)}{dt} = 2\gamma_{4,1} I_{ICU,0}(t,a,v_0,0,1) - 2\gamma_{4,1} I_{ICU,1}(t,a,v_0,0,1)$$

$$\frac{dI_{ICU,0}(t,a,v_0,1,1)}{dt} = \delta(ICU)(1-\mu(a))\phi_2(a)2\gamma_2 I_{CASE,1}(t,a,v_0) - 2\gamma_{4,1}I_{CCU,0}(t,a,v_0,1,1)$$

$$\frac{dI_{ICU,1}(t,a,v_0,1,1)}{dt} = 2\gamma_{4,1}I_{CCU,0}(t,a,v_0,1,1) - 2\gamma_{4,1}I_{ICU,1}(t,a,v_0,1,1)$$

$$\frac{dI_{REC,0}(t,a,v_0)}{dt} = 2\gamma_{4,1}I_{ICU,1}(t,a,v_0,0,1) + 2\gamma_{4,1}I_{ICU,1}(t,a,v_0,1,1) - 2\gamma_5 I_{REC,0}(t,a,v_0)$$

$$\frac{dI_{REC,1}(t,a,v_0)}{dt} = 2\gamma_5 I_{REC,0}(t,a,v_0) - 2\gamma_5 I_{REC,1}(t,a,v_0)$$

$$\frac{dR_1(t,a,v_0)}{dt} = \gamma_1 I_{MILD}(t,a,v_0) + 2\gamma_{3,1}I_{HOSPITAL,1}(t,a,v_0,0,1) + 2\gamma_{3,1}I_{HOSPITAL,1}(t,a,v_0,1,1) + 2\gamma_5 I_{REC,1}(t,a,v_0) + 2\gamma_{4,1}I_{ICU,1}(t,a,v_0,0,1) + 2\gamma_{4,1}I_{ICU,1}(t,a,v_0,1,1) - 2\rho R_1(t,a,v_0) - \kappa(a)R_1(t,a,v_0)$$

$$\frac{dR_2(t,a,v_0)}{dt} = 2\rho R_1(t,a,v_0) - 2\rho R_2(t,a,v_0) - \kappa(a)R_2(t,a,v_0)$$

$$\frac{dD(t,a,v_0)}{dt} = 2\gamma_{3,0}I_{HOSPITAL,1}(t,a,v_0,0,0) + 2\gamma_{3,0}I_{HOSPITAL,1}(t,a,v_0,1,0) + 2\gamma_{4,0}I_{ICU,1}(t,a,v_0,0,0) + 2\gamma_{4,0}I_{ICU,1}(t,a,v_0,1,0)$$

2. Vaccination group v1 (vaccinated but not yet protected (state 1))

$$\frac{dS(t,a,v_1)}{dt} = \kappa(a)S(t,a,v_0) + 2\rho R_2(t,a,v_1) - \beta\frac{S(t,a,v_1)}{N}\sum_{a'}c(a,a)\left[\sum_v\left(I_{MILD}(t,a',v) + I_{CASE}(t,a',v)\right)\right] - 2\omega S(t,a,v_1)$$

$$\frac{dE_1(t,a,v_1)}{dt} = \kappa(a)E_1(t,a,v_0) + \beta\frac{S(t,a,v_1)}{N}\sum_{a'}c(a,a)\left[\sum_v\left(I_{MILD}(t,a',v) + I_{CASE}(t,a',v)\right)\right] - 2\alpha E_1(t,a,v_1) - 2\omega E_1(t,a,v_1)$$

$$\frac{dE_2(t,a,v_1)}{dt} = \kappa(a)E_2(t,a,v_0) + 2\alpha E_1(t,a,v_1) - 2\alpha E_2(t,a,v_1) - 2\omega E_2(t,a,v_1)$$

$$\frac{dI_{MILD}(t,a,v_1)}{dt} = (1-\phi_1(a))\left(2\alpha E_2(t,a,v_1)\right) - \gamma_1 I_{MILD}(t,a,v_1) - 2\omega I_{MILD}(t,a,v_1)$$

$$\frac{dI_{CASE,0}(t,a,v_1)}{dt} = \phi_1(a)\left(2\alpha E_2(t,a,v_1)\right) - 2\gamma_2 I_{CASE,0}(t,a,v_1) - 2\omega I_{CASE,0}(t,a,v_1)$$

$$\frac{dI_{CASE,1}(t,a,v_1)}{dt} = 2\gamma_2 I_{CASE,0}(t,a,v_1) - 2\gamma_2 I_{CASE,1}(t,a,v_1) - 2\omega I_{CASE,1}(t,a,v_1)$$

$$\frac{dI_{HOSPITAL,0}(t,a,v_1,0,0)}{dt} = (1-\delta(H))\mu(a)(1-\phi_2(a))2\gamma_2 I_{CASE,1}(t,a,v_1) - 2\gamma_{3,0}I_{HOSPITAL,0}(t,a,v_1,0,0) - 2\omega I_{HOSPITAL,0}(t,a,v_1,0,0)$$

$$\frac{dI_{HOSPITAL,1}(t,a,v_1,0,0)}{dt} = 2\gamma_{3,0}I_{HOSPITAL,0}(t,a,v_1,0,0) - 2\gamma_{3,0}I_{HOSPITAL,1}(t,a,v_1,0,0) - 2\omega I_{HOSPITAL,1}(t,a,v_1,0,0)$$

$$\frac{dI_{HOSPITAL,0}(t,a,v_1,1{,}0)}{dt} = \delta(H)\mu(a)(1-\phi_2(a))2\gamma_2 I_{CASE,1}(t,a,v_1) - 2\gamma_{3,0} I_{HOSPITAL,0}(t,a,v_1,1{,}0) - 2\omega I_{HOSPITAL,0}(t,a,v_1,1{,}0)$$

$$\frac{dI_{HOSPITAL,1}(t,a,v_1,1{,}0)}{dt} = 2\gamma_{3,0} I_{HOSPITAL,0}(t,a,v_1,1{,}0) - 2\gamma_{3,0} I_{HOSPITAL,1}(t,a,v_1,1{,}0) - 2\omega I_{HOSPITAL,1}(t,a,v_1,1{,}0)$$

$$\frac{dI_{HOSPITAL,0}(t,a,v_1,0{,}1)}{dt} = (1-\delta(H))(1-\mu(a))(1-\phi_2(a))2\gamma_2 I_{CASE,1}(t,a,v_1) - 2\gamma_{3,1} I_{HOSPITAL,0}(t,a,v_1,0{,}1) - 2\omega I_{HOSPITAL\ ,0}(t,a,v_1,0{,}1)$$

$$\frac{dI_{HOSPITAL,1}(t,a,v_1,0{,}1)}{dt} = 2\gamma_{3,1} I_{HOSPITAL,0}(t,a,v_1,0{,}1) - 2\gamma_{3,1} I_{HOSPITAL,1}(t,a,v_1,0{,}1) - 2\omega I_{HOSPITAL,1}(t,a,v_1,0{,}1)$$

$$\frac{dI_{HOSPITAL,0}(t,a,v_1,1{,}1)}{dt} = \delta(H)(1-\mu(a))(1-\phi_2(a))2\gamma_2 I_{CASE,1}(t,a,v_1) - 2\gamma_{3,1} I_{HOSPITAL,0}(t,a,v_1,1{,}1) - 2\omega I_{HOSPITAL,0}(t,a,v_1,1{,}1)$$

$$\frac{dI_{HOSPITAL,1}(t,a,v_1,1{,}1)}{dt} = 2\gamma_{3,1} I_{HOSPITAL,0}(t,a,v_1,1{,}1) - 2\gamma_{3,1} I_{HOSPITAL,1}(t,a,v_1,1{,}1) - 2\omega I_{HOSPITAL,1}(t,a,v_1,1{,}1)$$

$$\frac{dI_{ICU,0}(t,a,v_1,0{,}0)}{dt} = (1-\delta(ICU))\mu(a)\phi_2(a)2\gamma_2 I_{CASE,1}(t,a,v_1) - 2\gamma_{4,0} I_{ICU,0}(t,a,v_1,0{,}0) - 2\omega I_{ICU,0}(t,a,v_1,0{,}0)$$

$$\frac{dI_{ICU,1}(t,a,v_1,0{,}0)}{dt} = 2\gamma_{4,0} I_{ICU,0}(t,a,v_1,0{,}0) - 2\gamma_{4,0} I_{ICU,1}(t,a,v_1,0{,}0) - 2\omega I_{ICU,1}(t,a,v_1,0{,}0)$$

$$\frac{dI_{ICU,0}(t,a,v_1,1{,}0)}{dt} = \delta(ICU)\mu(a)\phi_2(a)2\gamma_2 I_{CASE,1}(t,a,v_1) - 2\gamma_{4,0} I_{ICU,0}(t,a,v_1,1{,}0) - 2\omega I_{ICU,0}(t,a,v_1,1{,}0)$$

$$\frac{dI_{ICU,1}(t,a,v_1,1{,}0)}{dt} = 2\gamma_{4,0} I_{ICU,0}(t,a,v_1,1{,}0) - 2\gamma_{4,0} I_{ICU,1}(t,a,v_1,1{,}0) - 2\omega I_{ICU,1}(t,a,v_1,1{,}0)$$

$$\frac{dI_{ICU,0}(t,a,v_1,0{,}1)}{dt} = (1-\delta(ICU))(1-\mu(a))\phi_2(a)2\gamma_2 I_{CASE,1}(t,v_1,a) - 2\gamma_{4,1} I_{ICU,0}(t,a,v_1,0{,}1) - 2\omega I_{ICU,0}(t,a,v_1,0{,}1)$$

$$\frac{dI_{ICU,1}(t,a,v_1,0{,}1)}{dt} = 2\gamma_{4,1} I_{ICU,0}(t,a,v_1,0{,}1) - 2\gamma_{4,1} I_{ICU,1}(t,a,v_1,0{,}1) - 2\omega I_{ICU,1}(t,a,v_1,0{,}1)$$

$$\frac{dI_{ICU,0}(t,a,v_1,1{,}1)}{dt} = \delta(ICU)(1-\mu(a))\phi_2(a)2\gamma_2 I_{CASE,1}(t,a,v_1) - 2\gamma_{4,1} I_{ICU,0}(t,a,v_1,1{,}1) - 2\omega I_{ICU,0}(t,a,v_1,1{,}1)$$

$$\frac{dI_{ICU,1}(t,a,v_1,1{,}1)}{dt} = 2\gamma_{4,1} I_{ICU,0}(t,a,v_1,1{,}1) - 2\gamma_{4,1} I_{ICU,1}(t,a,v_1,1{,}1) - 2\omega I_{ICU,1}(t,a,v_1,1{,}1)$$

$$\frac{dI_{REC,0}(t,a,v_1)}{dt} = 2\gamma_{4,1}I_{ICU,1}(t,a,v_1,0{,}1) + 2\gamma_{4,1}I_{ICU,1}(t,a,v_1,1{,}1) - 2\gamma_5 I_{REC,0}(t,a,v_1) - 2\omega I_{REC,0}(t,a,v_1)$$

$$\frac{dI_{REC,1}(t,a,v_1)}{dt} = 2\gamma_5 I_{REC,0}(t,a,v_1) - 2\gamma_5 I_{REC,1}(t,a,v_1) - 2\omega I_{REC,1}(t,a,v_1)$$

$$\frac{dR_1(t,a,v_1)}{dt} = \kappa(a)R_1(t,a,v_0) + \gamma_1 I_{MILD}(t,a,v_1) + 2\gamma_{3,1}I_{HOSPITAL,1}(t,a,v_1,0{,}1) + 2\gamma_{3,1}I_{HOSPITAL,1}(t,a,v_1,1{,}1)$$
$$+ 2\gamma_5 I_{REC,1}(t,a,v_1) + 2\gamma_{4,1}I_{ICU,1}(t,a,v_1,0{,}1) + 2\gamma_{4,1}I_{ICU,1}(t,a,v_1,1{,}1) - 2\rho R_1(t,a,v_1)$$
$$- 2\omega R(t,a,v_1)$$

$$\frac{dR_2(t,a,v_1)}{dt} = \kappa(a)R_2(t,a,v_0) + 2\rho R_1(t,a,v_1) - 2\rho R_2(t,a,v_1)$$

$$\frac{dD(t,a,v_1)}{dt} = 2\gamma_{3,0}I_{HOSPITAL,1}(t,a,v_1,0{,}0) + 2\gamma_{3,0}I_{HOSPITAL,1}(t,a,v_1,1{,}0) + 2\gamma_{4,0}I_{ICU,1}(t,a,v_1,0{,}0)$$
$$+ 2\gamma_{4,0}I_{ICU,1}(t,a,v_1,1{,}0)$$

3. Vaccination group v_2 (vaccinated but not yet protected (state 2))

$$\frac{dS(t,a,v_2)}{dt} = 2\rho R_2(t,a,v_2) - \beta\frac{S(t,a,v_2)}{N}\textstyle\sum_{a'} c(a,a)\left[\sum_v \left(I_{MILD}(t,a',v) + I_{CASE}(t,a',v)\right)\right] + 2\omega S(t,a,v_1) - 2\omega S(t,a,v_2)$$

$$\frac{dE_1(t,a,v_2)}{dt} = \beta\frac{S(t,a,v_2)}{N}\textstyle\sum_{a'} c(a,a)\left[\sum_v \left(I_{MILD}(t,a',v) + I_{CASE}(t,a',v)\right)\right] - 2\alpha E_1(t,a,v_2) + 2\omega E_1(t,a,v_1)$$
$$- 2\omega E_1(t,a,v_2)$$

$$\frac{dE_2(t,a,v_2)}{dt} = 2\alpha E_1(t,a,v_2) - 2\alpha E_2(t,a,v_2) + 2\omega E_2(t,a,v_1) - 2\omega E_2(t,a,v_2)$$

$$\frac{dI_{MILD}(t,a,v_2)}{dt} = (1-\phi_1(a))\left(2\alpha E_2(t,a,v_2)\right) - \gamma_1 I_{MILD}(t,a,v_2) + 2\omega I_{MILD}(t,a,v_1) - 2\omega I_{MILD}(t,a,v_2)$$

$$\frac{dI_{CASE,0}(t,a,v_2)}{dt} = \phi_1(a)\left(2\alpha E_2(t,a,v_2)\right) - 2\gamma_2 I_{CASE,0}(t,a,v_2) + 2\omega I_{CASE,0}(t,a,v_1) - 2\omega I_{CASE,0}(t,a,v_2)$$

$$\frac{dI_{CASE,1}(t,a,v_2)}{dt} = 2\gamma_2 I_{CASE,0}(t,a,v_2) - 2\gamma_2 I_{CASE,1}(t,a,v_2) + 2\omega I_{CASE,1}(t,a,v_1) - 2\omega I_{CASE,1}(t,a,v_2)$$

$$\frac{dI_{HOSPITAL,0}(t,a,v_2,0{,}0)}{dt}$$
$$= (1-\delta(H))\mu(a)(1-\phi_2(a))2\gamma_2 I_{CASE,1}(t,a,v_2) - 2\gamma_{3,0}I_{HOSPITAL,0}(t,a,v_2,0{,}0)$$
$$+ 2\omega I_{HOSPITAL,0}(t,a,v_1,0{,}0) - 2\omega I_{HOSPITAL,0}(t,a,v_2,0{,}0)$$

$$\frac{dI_{\mathrm{HOSPITAL,\,1}}(t,a,v_2,0{,}0)}{dt}$$
$$= 2\gamma_{3,0}I_{\mathrm{HOSPITAL,\,0}}(t,a,v_2,0{,}0) - 2\gamma_{3,0}I_{\mathrm{HOSPITAL,}}(t,a,v_2,0{,}0) + 2\omega I_{\mathrm{HOSPITAL\,,1}}(t,a,v_1,0{,}0)$$
$$- 2\omega I_{HOSPITAL,1}(t,a,v_2,0{,}0)$$

$$\frac{dI_{HOSPITAL,0}(t,a,v_2,1{,}0)}{dt}$$
$$= \delta(H)\mu(a)(1-\phi_2(a))2\gamma_2 I_{CASE,1}(t,a,v_2) - 2\gamma_{3,0}I_{HOSPITAL,0}(t,a,v_2,1{,}0) + 2\omega I_{HOSPITAL,0}(t,a,v_1,1{,}0)$$
$$- 2\omega I_{HOSPITAL,0}(t,a,v_2,1{,}0)$$

$$\frac{dI_{HOSPITAL,1}(t,a,v_2,1,0)}{dt} = 2\gamma_{3,0}I_{HOSPITAL,0}(t,a,v_2,1,0) - 2\gamma_{3,0}I_{HOSPITAL,1}(t,a,v_2,1,0) + 2\omega I_{HOSPITAL,1}(t,a,v_1,1,0) - 2\omega I_{HOSPITAL,1}(t,a,v_2,1,0)$$

$$\frac{dI_{HOSPITAL,0}(t,a,v_2,0,1)}{dt} = (1-\delta(H))(1-\mu(a))(1-\phi_2(a))2\gamma_2 I_{CASE,1}(t,a,v_2) - 2\gamma_{3,1}I_{HOSPITAL,0}(t,a,v_2,0,1) + 2\omega I_{HOSPITAL,0}(t,a,v_1,0,1) - 2\omega I_{HOSPITAL,0}(t,a,v_2,0,1)$$

$$\frac{dI_{HOSPITAL,1}(t,a,v_2,0,1)}{dt} = 2\gamma_{3,1}I_{HOSPITAL,0}(t,a,v_2,0,1) - 2\gamma_{3,1}I_{HOSPITAL,1}(t,a,v_2,0,1) + 2\omega I_{HOSPITAL,1}(t,a,v_1,0,1) - 2\omega I_{HOSPITAL,1}(t,a,v_2,0,1)$$

$$\frac{dI_{HOSPITAL,0}(t,a,v_2,1,1)}{dt} = \delta(H)(1-\mu(a))(1-\phi_2(a))2\gamma_2 I_{CASE,1}(t,a,v_2) - 2\gamma_{3,1}I_{HOSPITAL,0}(t,a,v_2,1,1) + 2\omega I_{HOSPITAL,0}(t,a,v_1,1,1) - 2\omega I_{HOSPITAL,0}(t,a,v_2,1,1)$$

$$\frac{dI_{HOSPITAL,1}(t,a,v_2,1,1)}{dt} = 2\gamma_{3,1}I_{HOSPITAL,0}(t,a,v_2,1,1) - 2\gamma_{3,1}I_{HOSPITAL,1}(t,a,v_2,1,1) + 2\omega I_{HOSPITAL,1}(t,a,v_1,1,1) - 2\omega I_{HOSPITAL,1}(t,a,v_2,1,1)$$

$$\frac{dI_{ICU,0}(t,a,v_2,0,0)}{dt} = (1-\delta(ICU))\mu(a)\phi_2(a)2\gamma_2 I_{CASE,1}(t,a,v_2) - 2\gamma_{4,0}I_{ICU,0}(t,a,v_2,0,0) + 2\omega I_{ICU,0}(t,a,v_1,0,0) - 2\omega I_{ICU,0}(t,a,v_2,0,0)$$

$$\frac{dI_{ICU,1}(t,a,v_2,0,0)}{dt} = 2\gamma_{4,0}I_{ICU,0}(t,a,v_2,0,0) - 2\gamma_{4,0}I_{ICU,1}(t,a,v_2,0,0) + 2\omega I_{ICU,1}(t,a,v_1,0,0) - 2\omega I_{ICU,1}(t,a,v_2,0,0)$$

$$\frac{dI_{ICU,0}(t,a,v_2,1,0)}{dt} = \delta(ICU)\mu(a)\phi_2(a)2\gamma_2 I_{CASE,1}(t,a,v_2) - 2\gamma_{4,0}I_{ICU,0}(t,a,v_2,1,0) + 2\omega I_{ICU,0}(t,a,v_1,1,0) - 2\omega I_{ICU,0}(t,a,v_2,1,0)$$

$$\frac{dI_{ICU,1}(t,a,v_2,1,0)}{dt} = 2\gamma_{4,0}I_{ICU,0}(t,a,v_2,1,0) - 2\gamma_{4,0}I_{ICU,1}(t,a,v_2,1,0) + 2\omega I_{ICU,1}(t,a,v_1,1,0) - 2\omega I_{ICU,1}(t,a,v_2,1,0)$$

$$\frac{dI_{ICU,0}(t,a,v_2,0,1)}{dt} = (1-\delta(ICU))(1-\mu(a))\phi_2(a)2\gamma_2 I_{CASE,1}(t,v_2,a) - 2\gamma_{4,1}I_{ICU,0}(t,a,v_2,0,1) + 2\omega I_{ICU,0}(t,a,v_1,0,1) - 2\omega I_{ICU,0}(t,a,v_2,0,1)$$

$$\frac{dI_{ICU,1}(t,a,v_2,0,1)}{dt} = 2\gamma_{4,1}I_{ICU,0}(t,a,v_2,0,1) - 2\gamma_{4,1}I_{ICU,1}(t,a,v_2,0,1) + 2\omega I_{ICU,1}(t,a,v_1,0,1) - 2\omega I_{ICU,1}(t,a,v_2,0,1)$$

$$\frac{dI_{ICU,0}(t,a,v_2,1,1)}{dt} = \delta(ICU)(1-\mu(a))\phi_2(a)2\gamma_2 I_{CASE,1}(t,a,v_2) - 2\gamma_{4,1}I_{ICU,0}(t,a,v_2,1,1) + 2\omega I_{ICU,0}(t,a,v_1,1,1) - 2\omega I_{ICU,0}(t,a,v_2,1,1)$$

$$\frac{dI_{ICU,1}(t,a,v_2,1,1)}{dt} = 2\gamma_{4,1}I_{ICU,0}(t,a,v_2,1,1) - 2\gamma_{4,1}I_{ICU,1}(t,a,v_2,1,1) + 2\omega I_{ICU,1}(t,a,v_1,1,1) - 2\omega I_{ICU,1}(t,a,v_2,1,1)$$

$$\frac{dI_{REC,0}(t,a,v_2)}{dt} = 2\gamma_{4,1}I_{ICU,1}(t,a,v_2,0,1) + 2\gamma_{4,1}I_{ICU,1}(t,a,v_2,1,1) - 2\gamma_5 I_{REC,0}(t,a,v_2) + 2\omega I_{REC,0}(t,a,v_1) - 2\omega I_{REC,0}(t,a,v_2)$$

$$\frac{dI_{REC,1}(t,a,v_2)}{dt} = 2\gamma_5 I_{REC,0}(t,a,v_2) - 2\gamma_5 I_{REC,1}(t,a,v_2) + 2\omega I_{REC,1}(t,a,v_1) - 2\omega I_{REC,1}(t,a,v_2)$$

$$\frac{dR_1(t,a,v_2)}{dt} = \gamma_1 I_{MILD}(t,a,v_2) + 2\gamma_{3,1}I_{HOSPITAL,1}(t,a,v_2,0,1) + 2\gamma_{3,1}I_{HOSPITAL,1}(t,a,v_2,1,1) + 2\gamma_5 I_{REC,1}(t,a,v_2) + 2\gamma_{4,1}I_{ICU,1}(t,a,v_2,0,1) + 2\gamma_{4,1}I_{ICU,1}(t,a,v_2,1,1) - 2\rho R_1(t,a,v_2) + 2\omega R_1(t,a,v_1) - 2\omega R_1(t,a,v_2)$$

$$\frac{dR_2(t,a,v_2)}{dt} = 2\rho R_1(t,a,v_2) - 2\rho R_2(t,a,v_2) + 2\omega R_2(t,a,v_1) - 2\omega R_2(t,a,v_2)$$

$$\frac{dD(t,a,v_2)}{dt} = 2\gamma_{3,0}I_{HOSPITAL,}(t,a,v_2,0,0) + 2\gamma_{3,0}I_{HOSPITAL\,,1}(t,a,v_2,1,0) + 2\gamma_{4,0}I_{ICU,1}(t,a,v_2,0,0) + 2\gamma_{4,0}I_{ICU,1}(t,a,v_2,1,0)$$

4. Vaccination group v3 (vaccinated and protected (state 1))

$$\frac{dS(t,a,v_3)}{dt} = 2\rho R_2(t,a,v_3) - v_{inf}(a)\beta\frac{S(t,a,v_3)}{N}\sum_{a'} c(a,a')\left[\sum_v \left(I_{MILD}(t,a',v) + I_{CASE}(t,a',v)\right)\right] + 2\omega S(t,a,v_2) - 2\psi S(t,a,v_3)$$

$$\frac{dE_1(t,a,v_3)}{dt} = v_{inf}(a)\beta\frac{S(t,a,v_3)}{N}\sum_{a'} c(a,a)\left[\sum_v \left(I_{MILD}(t,a',v) + I_{CASE}(t,a',v)\right)\right] - 2\alpha E_1(t,a,v_2) + 2\omega E_1(t,a,v_2) - 2\psi E_1(t,a,v_3)$$

$$\frac{dE_2(t,a,v_3)}{dt} = 2\alpha E_1(t,a,v_3) - 2\alpha E_2(t,a,v_3) + 2\omega E_2(t,a,v_2) - 2\psi E_2(t,a,v_3)$$

$$\frac{dI_{MILD}(t,a,v_3)}{dt} = (1 - v_{dis}(a)\phi_1(a))\left(2\alpha E_2(t,a,v_3)\right) - \gamma_1 I_{MILD}(t,a,v_3) + 2\omega I_{MILD}(t,a,v_2) - 2\psi I_{MILD}(t,a,v_3)$$

$$\frac{dI_{CASE,0}(t,a,v_3)}{dt} = v_{dis}(a)\phi_1(a)\left(2\alpha E_2(t,a,v_3)\right) - 2\gamma_2 I_{CASE,0}(t,a,v_3) + 2\omega I_{CASE,0}(t,a,v_2) - 2\psi I_{CASE,0}(t,a,v_3)$$

$$\frac{dI_{CASE,1}(t,a,v_3)}{dt} = 2\gamma_2 I_{CASE,0}(t,a,v_3) - 2\gamma_2 I_{CASE,1}(t,a,v_3) + 2\omega I_{CASE,1}(t,a,v_2) - 2\psi I_{CASE,1}(t,a,v_3)$$

$$\frac{dI_{HOSPITAL,0}(t,a,v_3,0,0)}{dt} = (1 - \delta(H))\mu(a)(1 - \phi_2(a))2\gamma_2 I_{CASE,1}(t,a,v_3) - 2\gamma_{3,0}I_{HOSPITAL,0}(t,a,v_3,0,0) + 2\omega I_{HOSPITAL,0}(t,a,v_2,0,0) - 2\psi I_{HOSPITAL,0}(t,a,v_3,0,0)$$

$$\frac{dI_{HOSPITAL,\,1}(t,a,v_3,0,0)}{dt} = 2\gamma_{3,0}I_{HOSPITAL,0}(t,a,v_3,0,0) - 2\gamma_{3,0}I_{HOSPITAL,1}(t,a,v_3,0,0) + 2\omega I_{HOSPITAL,1}(t,a,v_2,0,0) - 2\psi I_{HOSPITAL\,,1}(t,a,v_3,0,0)$$

$$\frac{dI_{HOSPITAL,0}(t,a,v_3,1,0)}{dt} = \delta(H)\mu(a)(1 - \phi_2(a))2\gamma_2 I_{CASE,1}(t,a,v_3) - 2\gamma_{3,0}I_{HOSPITAL,0}(t,a,v_3,1,0) + 2\omega I_{HOSPITAL,0}(t,a,v_2,1,0) - 2\psi_{HOSPITAL\,,0}(t,a,v_3,1,0)$$

$$\frac{dI_{HOSPITAL,1}(t,a,v_3,1,0)}{dt} = 2\gamma_{3,0}I_{HOSPITAL,0}(t,a,v_3,1,0) - 2\gamma_{3,0}I_{HOSPITAL,1}(t,a,v_3,1,0) + 2\omega I_{HOSPITAL,1}(t,a,v_2,1,0) - 2\psi I_{HOSPITAL,1}(t,a,v_3,1,0)$$

$$\frac{dI_{HOSPITAL,0}(t,a,v_3,0,1)}{dt} = (1-\delta(H))(1-\mu(a))(1-\phi_2(a))2\gamma_2 I_{CASE,1}(t,a,v_3) - 2\gamma_{3,1}I_{HOSPITAL,0}(t,a,v_3,0,1) + 2\omega I_{HOSPITAL,0}(t,a,v_2,0,1) - 2\psi I_{HOSPITAL,0}(t,a,v_3,0,1)$$

$$\frac{dI_{HOSPITAL,1}(t,a,v_3,0,1)}{dt} = 2\gamma_{3,1}I_{HOSPITAL\ 0}(t,a,v_3,0,1) - 2\gamma_{3,1}I_{HOSPITAL,}(t,a,v_3,0,1) + 2\omega I_{HOSPITAL\ ,1}(t,a,v_2,0,1) - 2\psi I_{HOSPITAL,1}(t,a,v_3,0,1)$$

$$\frac{dI_{HOSPITAL,0}(t,a,v_3,1,1)}{dt} = \delta(H)(1-\mu(a))(1-\phi_2(a))2\gamma_2 I_{CASE,1}(t,a,v_3) - 2\gamma_{3,1}I_{HOSPITAL,0}(t,a,v_3,1,1)2\omega I_{HOSPITAL,\ 0}(t,a,v_2,1,1) - 2\psi I_{HOSPITAL\ ,0}(t,a,v_3,1,1)$$

$$\frac{dI_{\mathrm{HOSPITAL},\ 1}(t,a,v_3,1,1)}{dt} = 2\gamma_{3,1}I_{HOSPITAL,0}(t,a,v_3,1,1) - 2\gamma_{3,1}I_{HOSPITAL,1}(t,a,v_3,1,1) + 2\omega I_{HOSPITAL,1}(t,a,v_2,1,1) - 2\psi I_{HOSPITAL,1}(t,a,v_3,1,1)$$

$$\frac{dI_{ICU,0}(t,a,v_3,0,0)}{dt} = (1-\delta(ICU))\mu(a)\phi_2(a)2\gamma_2 I_{CASE,1}(t,a,v_3) - 2\gamma_{4,0}I_{ICU,0}(t,a,v_3,0,0) + 2\omega I_{ICU,0}(t,a,v_2,0,0) - 2\psi I_{ICU,0}(t,a,v_3,0,0)$$

$$\frac{dI_{ICU,1}(t,a,v_3,0,0)}{dt} = 2\gamma_{4,0}I_{ICU,0}(t,a,v_3,0,0) - 2\gamma_{4,0}I_{ICU,1}(t,a,v_3,0,0) + 2\omega I_{ICU,1}(t,a,v_2,0,0) - 2\psi I_{ICU,1}(t,a,v_3,0,0)$$

$$\frac{dI_{ICU,0}(t,a,v_3,1,0)}{dt} = \delta(ICU)\mu(a)\phi_2(a)2\gamma_2 I_{CASE,1}(t,a,v_3) - 2\gamma_{4,0}I_{ICU,0}(t,a,v_3,1,0) + 2\omega I_{ICU,0}(t,a,v_2,1,0) - 2\psi I_{ICU,0}(t,a,v_3,1,0)$$

$$\frac{dI_{ICU,1}(t,a,v_3,1,0)}{dt} = 2\gamma_{4,0}I_{ICU,0}(t,a,v_3,1,0) - 2\gamma_{4,0}I_{ICU,1}(t,a,v_3,1,0) + 2\omega I_{ICU,1}(t,a,v_2,1,0) - 2\psi I_{ICU,1}(t,a,v_3,1,0)$$

$$\frac{dI_{ICU,0}(t,a,v_3,0,1)}{dt} = (1-\delta(ICU))(1-\mu(a))\phi_2(a)2\gamma_2 I_{CASE,1}(t,v_3,a) - 2\gamma_{4,1}I_{ICU,0}(t,a,v_3,0,1) + 2\omega I_{ICU,0}(t,a,v_2,0,1) - 2\psi I_{ICU,0}(t,a,v_3,0,1)$$

$$\frac{dI_{ICU,1}(t,a,v_3,0,1)}{dt} = 2\gamma_{4,1}I_{ICU,0}(t,a,v_3,0,1) - 2\gamma_{4,1}I_{ICU,1}(t,a,v_3,0,1) + 2\omega I_{ICU,1}(t,a,v_2,0,1) - 2\psi I_{ICU,1}(t,a,v_3,0,1)$$

$$\frac{dI_{ICU,0}(t,a,v_3,1,1)}{dt} = \delta(ICU)(1-\mu(a))\phi_2(a)2\gamma_2 I_{CASE,1}(t,a,v_3) - 2\gamma_{4,1}I_{ICU,0}(t,a,v_3,1,1) + 2\omega I_{ICU,0}(t,a,v_2,1,1) - 2\psi I_{ICU,0}(t,a,v_3,1,1)$$

$$\frac{dI_{ICU,1}(t,a,v_3,1,1)}{dt} = 2\gamma_{4,1}I_{ICU,0}(t,a,v_3,1,1) - 2\gamma_{4,1}I_{ICU,1}(t,a,v_3,1,1) + 2\omega I_{ICU,1}(t,a,v_2,1,1) - 2\psi I_{ICU,1}(t,a,v_3,1,1)$$

$$\frac{dI_{REC,0}(t,a,v_3)}{dt} = 2\gamma_{4,1}I_{ICU,1}(t,a,v_3,0,1) + 2\gamma_{4,1}I_{ICU,1}(t,a,v_3,1,1) - 2\gamma_5 I_{REC,0}(t,a,v_3) + 2\omega I_{REC,0}(t,a,v_2) - 2\psi I_{REC,0}(t,a,v_3)$$

$$\frac{dI_{REC,1}(t,a,v_3)}{dt} = 2\gamma_5 I_{REC,0}(t,a,v_3) - 2\gamma_5 I_{REC,1}(t,a,v_3) + 2\omega I_{REC,1}(t,a,v_2) - 2\psi I_{REC,1}(t,a,v_3)$$

$$\frac{dR_1(t,a,v_3)}{dt} = \gamma_1 I_{MILD}(t,a,v_3) + 2\gamma_{3,1}I_{HOSPITAL,1}(t,a,v_3,0,1) + 2\gamma_{3,1}I_{HOSPITAL,1}(t,a,v_3,1,1) + 2\gamma_5 I_{REC,1}(t,a,v_3) + 2\gamma_{4,1}I_{ICU,1}(t,a,v_3,0,1) + 2\gamma_{4,1}I_{ICU,1}(t,a,v_3,1,1) - 2\rho R_1(t,a,v_3) + 2\omega R_1(t,a,v_2) - 2\psi R_1(t,a,v_3)$$

$$\frac{dR_2(t,a,v_3)}{dt} = 2\rho R_1(t,a,v_3) - 2\rho R_2(t,a,v_3) + 2\omega R_2(t,a,v_2) - 2\psi R_2(t,a,v_3)$$

$$\frac{dD(t,a,v_3)}{dt} = 2\gamma_{3,0}I_{HOSPITAL,1}(t,a,v_3,0,0) + 2\gamma_{3,0}I_{HOSPITAL,1}(t,a,v_3,1,0) + 2\gamma_{4,0}I_{ICU,1}(t,a,v_3,0,0) + 2\gamma_{4,0}I_{ICU,1}(t,a,v_3,1,0)$$

5. Vaccination group v4 (vaccinated and protected (state 2))

$$\frac{dS(t,a,v_4)}{dt} = 2\rho R_2(t,a,v_4) - v_{inf}(a)\beta\frac{S(t,a,v_4)}{N}\sum_{a'} c(a,a)\left[\sum_v \left(I_{MILD}(t,a',v) + I_{CASE}(t,a',v)\right)\right] + 2\psi S(t,a,v_3) - 2\psi S(t,a,v_4)$$

$$\frac{dI_{HOSPITAL,1}(t,a,v_4,0,0)}{dt} = 2\gamma_{3,0}I_{HOSPITAL,0}(t,a,v_4,0,0) - 2\gamma_{3,0}I_{HOSPITAL,1}(t,a,v_4,0,0) + 2\psi I_{HOSPITAL,1}(t,a,v_3,0,0) - 2\psi I_{\mathrm{HOSPITAL},1}(t,a,v_4,0,0)$$

$$\frac{dI_{HOSPITAL,0}(t,a,v_4,1,0)}{dt} = \delta(H)\mu(a)(1-\phi_2(a))2\gamma_2 I_{CASE,1}(t,a,v_4) - 2\gamma_{3,0}I_{HOSPITAL,0}(t,a,v_4,1,0) + 2\psi I_{HOSPITAL,0}(t,a,v_3,1,0) - 2\psi I_{\mathrm{HOSPITAL},0}(t,a,v_3,1,0)$$

$$\frac{dI_{HOSPITAL,1}(t,a,v_4,1,0)}{dt} = 2\gamma_{3,0}I_{HOSPITAL,0}(t,a,v_4,1,0) - 2\gamma_{3,0}I_{HOSPITAL,1}(t,a,v_4,1,0) + 2\psi I_{HOSPITAL,1}(t,a,v_3,1,0) - 2\psi I_{\mathrm{HOSPITAL},1}(t,a,v_4,1,0)$$

$$\frac{dI_{HOSPITAL,0}(t,a,v_4,0,1)}{dt} = (1-\delta(H))(1-\mu(a))(1-\phi_2(a))2\gamma_2 I_{CASE,1}(t,a,v_4) - 2\gamma_{3,1}I_{HOSPITAL,0}(t,a,v_4,0,1) + 2\psi I_{HOSPITAL,0}(t,a,v_3,0,1) - 2\psi I_{HOSPITAL,0}(t,a,v_4,0,1)$$

$$\frac{dI_{HOSPITAL,1}(t,a,v_4,0,1)}{dt} = 2\gamma_{3,1}I_{HOSPITAL,0}(t,a,v_4,0,1) - 2\gamma_{3,1}I_{HOSPITAL,1}(t,a,v_4,0,1) + 2\psi I_{HOSPITAL,1}(t,a,v_3,0,1) - 2\psi I_{HOSPITAL,1}(t,a,v_4,0,1)$$

$$\frac{dI_{HOSPITAL,0}(t,a,v_4,1,1)}{dt} = \delta(H)(1-\mu(a))(1-\phi_2(a))2\gamma_2 I_{CASE,1}(t,a,v_4) - 2\gamma_{3,1}I_{HOSPITAL,0}(t,a,v_4,1,1) + 2\psi I_{HOSPITAL,0}(t,a,v_3,1,1) - 2\psi I_{HOSPITAL,0}(t,a,v_4,1,1)$$

$$\frac{dI_{HOSPITAL,1}(t,a,v_4,1,1)}{dt} = 2\gamma_{3,1}I_{HOSPITAL,0}(t,a,v_4,1,1) - 2\gamma_{3,1}I_{HOSPITAL,1}(t,a,v_4,1,1) + 2\psi I_{HOSPITAL,1}(t,a,v_3,1,1) - 2\psi I_{HOSPITAL\ ,1}(t,a,v_4,1,1)$$

$$\frac{dI_{ICU,0}(t,a,v_4,0,0)}{dt} = (1-\delta(ICU))\mu(a)\phi_2(a)2\gamma_2 I_{CASE,1}(t,a,v_4) - 2\gamma_{4,0}I_{ICU,0}(t,a,v_4,0,0) + 2\psi I_{ICU,0}(t,a,v_3,0,0) - 2\psi I_{ICU,0}(t,a,v_4,0,0)$$

$$\frac{dI_{ICU,1}(t,a,v_4,0,0)}{dt} = 2\gamma_{4,0}I_{ICU,0}(t,a,v_4,0,0) - 2\gamma_{4,0}I_{ICU,1}(t,a,v_4,0,0) + 2\psi I_{ICU,1}(t,a,v_3,0,0) - 2\psi I_{ICU,1}(t,a,v_4,0,0)$$

$$\frac{dI_{ICU,0}(t,a,v_4,1,0)}{dt} = \delta(ICU)\mu(a)\phi_2(a)2\gamma_2 I_{CASE,1}(t,a,v_4) - 2\gamma_{4,0}I_{ICU,0}(t,a,v_4,1,0) + 2\psi I_{ICU,0}(t,a,v_3,1,0) - 2\psi I_{ICU,0}(t,a,v_4,1,0)$$

$$\frac{dI_{ICU,1}(t,a,v_4,1,0)}{dt} = 2\gamma_{4,0}I_{ICU,0}(t,a,v_4,1,0) - 2\gamma_{4,0}I_{ICU,1}(t,a,v_4,1,0) + 2\psi I_{ICU,1}(t,a,v_3,1,0) - 2\psi I_{ICU,1}(t,a,v_4,1,0)$$

$$\frac{dI_{ICU,0}(t,a,v_4,0,1)}{dt} = (1-\delta(ICU))(1-\mu(a))\phi_2(a)2\gamma_2 I_{CASE,1}(t,v_4,a) - 2\gamma_{4,1}I_{ICU,0}(t,a,v_4,0,1) + 2I_{ICU,0}(t,a,v_3,0,1) - 2\psi I_{ICU,0}(t,a,v_4,0,1)$$

$$\frac{dI_{ICU,1}(t,a,v_4,0,1)}{dt} = 2\gamma_{4,1}I_{ICU,0}(t,a,v_4,0,1) - 2\gamma_{4,1}I_{ICU,1}(t,a,v_4,0,1) + 2\psi I_{ICU,1}(t,a,v_3,0,1) - 2\psi I_{ICU,1}(t,a,v_4,0,1)$$

$$\frac{dI_{ICU,0}(t,a,v_4,1,1)}{dt} = \delta(ICU)(1-\mu(a))\phi_2(a)2\gamma_2 I_{CASE,1}(t,a,v_4) - 2\gamma_{4,1}I_{ICU,0}(t,a,v_4,1,1) + 2\psi I_{ICU,0}(t,a,v_3,1,1) - 2\psi I_{ICU,0}(t,a,v_4,1,1)$$

$$\frac{dI_{ICU,1}(t,a,v_4,1,1)}{dt} = 2\gamma_{4,1}I_{ICU,0}(t,a,v_4,1,1) - 2\gamma_{4,1}I_{ICU,1}(t,a,v_4,1,1) + 2\psi I_{ICU,1}(t,a,v_3,1,1) - 2\psi I_{ICU,1}(t,a,v_4,1,1)$$

$$\frac{dI_{REC,0}(t,a,v_4)}{dt} = 2\gamma_{4,1}I_{ICU,1}(t,a,v_4,0,1) + 2\gamma_{4,1}I_{ICU,1}(t,a,v_4,1,1) - 2\gamma_5 I_{REC,0}(t,a,v_4) + 2\psi I_{REC,0}(t,a,v_3) - 2\psi I_{REC,0}(t,a,v_4)$$

$$\frac{dI_{REC,1}(t,a,v_4)}{dt} = 2\gamma_5 I_{REC,0}(t,a,v_4) - 2\gamma_5 I_{REC,1}(t,a,v_4) + 2\psi I_{REC,1}(t,a,v_3) - 2\psi I_{REC,1}(t,a,v_4)$$

$$\frac{dR_1(t,a,v_4)}{dt} = \gamma_1 I_{MILD}(t,a,v_4) + 2\gamma_{3,1}I_{HOSPITAL,1}(t,a,v_4,0,1) + 2\gamma_{3,1}I_{HOSPITAL,1}(t,a,v_4,1,1) + 2\gamma_5 I_{REC,1}(t,a,v_4) + 2\gamma_{4,1}I_{ICU,1}(t,a,v_4,0,1) + 2\gamma_{4,1}I_{ICU,1}(t,a,v_4,1,1) - 2\rho R_1(t,a,v_4) + 2\psi R_1(t,a,v_3) - 2\psi R_1(t,a,v_4)$$

$$\frac{dR_2(t,a,v_4)}{dt} = 2\rho R_1(t,a,v_4) - 2\rho R_2(t,a,v_4) + 2\psi R_2(t,a,v_3) - 2\psi R_2(t,a,v_4)$$

$$\frac{dD(t,a,v_4)}{dt} = 2\gamma_{3,0}I_{HOSPITAL,1}(t,a,v_4,0,0) + 2\gamma_{3,0}I_{HOSPITAL,1}(t,a,v_4,1,0) + 2\gamma_{4,0}I_{ICU,1}(t,a,v_4,0,0) + 2\gamma_{4,0}I_{ICU,1}(t,a,v_4,1,0)$$

6. Vaccination group v5 (previously vaccinated but no longer protected)

$$\frac{dS(t,a,v_5)}{dt} = 2\rho R_2(t,a,v_5) - \beta \frac{S(t,a,v_5)}{N} \sum_{a'} c(a,a) \left[\sum_v \left(I_{MILD}(t,a',v) + I_{CASE}(t,a',v)\right)\right] + 2\psi S(t,a,v_4)$$

$$\frac{dE_1(t,a,v_5)}{dt} = \beta \frac{S(t,a,v_5)}{N} \sum_{a'} c(a,a) \left[\sum_v \left(I_{MIDD}(t,a',v) + I_{CASE}(t,a',v)\right)\right] - 2\alpha E_1(t,a,v_5) + 2\psi E_1(t,a,v_4)$$

$$\frac{dE_2(t,a,v_5)}{dt} = 2\alpha E_1(t,a,v_5) - 2\alpha E_2(t,a,v_5) + 2\psi E_2(t,a,v_4)$$

$$\frac{dI_{MILD}(t,a,v_5)}{dt} = (1-\phi_1(a))\left(2\alpha E_2(t,a,v_5)\right) - \gamma_1 I_{MILD}(t,a,v_5) + 2\psi I_{MILD}(t,a,v_4)$$

$$\frac{dI_{CASE,0}(t,a,v_5)}{dt} = \phi_1(a)\left(2\alpha E_2(t,a,v_5)\right) - 2\gamma_2 I_{CASE,0}(t,a,v_5) + 2\psi I_{CASE,0}(t,a,v_4)$$

$$\frac{dI_{CASE,1}(t,a,v_5)}{dt} = 2\gamma_2 I_{CASE,0}(t,a,v_5) - 2\gamma_2 I_{CASE,1}(t,a,v_5) + 2\psi I_{CASE,1}(t,a,v_4)$$

$$\frac{dI_{HOSPITAL,0}(t,a,v_5,0,0)}{dt} = (1-\delta(H))\mu(a)(1-\phi_2(a))2\gamma_2 I_{CASE,1}(t,a,v_5) - 2\gamma_{3,0} I_{HOSPITAL,0}(t,a,v_5,0,0) + 2\psi I_{HOSPITAL,0}(t,a,v_4,0,0)$$

$$\frac{dI_{HOSPITAL,1}(t,a,v_5,0,0)}{dt} = 2\gamma_{3,0} I_{HOSPITAL,0}(t,a,v_5,0,0) - 2\gamma_{3,0} I_{HOSPITAL,1}(t,a,v_5,0,0) + 2\psi I_{HOSPITAL,1}(t,a,v_4,0,0)$$

$$\frac{dI_{\text{HOSPITAL},0}(t,a,v_5,1,0)}{dt} = \delta(H)\mu(a)(1-\phi_2(a))2\gamma_2 I_{CASE,1}(t,a,v_5) - 2\gamma_{3,0} I_{HOSPITAL,0}(t,a,v_5,1,0) + 2\psi I_{HOSPITAL,0}(t,a,v_3,1,0)$$

$$\frac{dI_{HOSPITAL,1}(t,a,v_5,1,0)}{dt} = 2\gamma_{3,0} I_{HOSPITAL,0}(t,a,v_5,1,0) - 2\gamma_{3,0} I_{HOSPITAL,1}(t,a,v_5,1,0) + 2\psi I_{HOSPITAL,1}(t,a,v_4,1,0)$$

$$\frac{dI_{HOSPITAL,0}(t,a,v_5,0,1)}{=} (1-\delta(H))(1-\mu(a))(1-\phi_2(a))2\gamma_2 I_{CASE,1}(t,a,v_5) - 2\gamma_{3,1} I_{HOSPITAL,0}(t,a,v_5,0,1) + 2\psi I_{HOSPITAL,0}(t,a,v_4,0,1)$$

$$\frac{dI_{HOSPITAL,1}(t,a,v_5,0,1)}{dt} = 2\gamma_{3,1} I_{HOSPITAL,0}(t,a,v_5,0,1) - 2\gamma_{3,1} I_{HOSPITAL,1}(t,a,v_5,0,1) + 2\psi I_{HOSPITAL,1}(t,a,v_4,0,1)$$

$$\frac{dI_{\text{HOSPITAL, 0}}(t,a,v_5,1,1)}{dt} = \delta(H)(1-\mu(a))(1-\phi_2(a))2\gamma_2 I_{CASE,1}(t,a,v_5) - 2\gamma_{3,1} I_{HOSPITAL,0}(t,a,v_5,1,1) + 2\psi I_{HOSPITAL,0}(t,a,v_4,1,1)$$

$$\frac{dI_{\text{HOSPITAL, 1}}(t,a,v_5,1,1)}{dt} = 2\gamma_{3,1} I_{HOSPITAL,0}(t,a,v_5,1,1) - 2\gamma_{3,1} I_{HOSPITAL,1}(t,a,v_5,1,1) + 2\psi I_{HOSPITAL,1}(t,a,v_4,1,1)$$

$$\frac{dI_{ICU,0}(t,a,v_5,0,0)}{dt} = (1-\delta(ICU))\mu(a)\phi_2(a)2\gamma_2 I_{CASE,1}(t,a,v_5) - 2\gamma_{4,0} I_{ICU,0}(t,a,v_5,0,0) + 2\psi I_{ICU,0}(t,a,v_4,0,0)$$

$$\frac{dI_{ICU,1}(t,a,v_5,0,0)}{dt} = 2\gamma_{4,0} I_{ICU,0}(t,a,v_5,0,0) - 2\gamma_{4,0} I_{ICU,1}(t,a,v_5,0,0) + 2\psi I_{ICU,1}(t,a,v_4,0,0)$$

$$\frac{dI_{ICU,0}(t,a,v_5,1,0)}{dt} = \delta(ICU)\mu(a)\phi_2(a)2\gamma_2 I_{CASE,1}(t,a,v_5) - 2\gamma_{4,0} I_{ICU,0}(t,a,v_5,1,0) + 2\psi I_{ICU,0}(t,a,v_4,1,0)$$

$$\frac{dI_{ICU,1}(t,a,v_5,1,0)}{dt} = 2\gamma_{4,0} I_{ICU,0}(t,a,v_5,1,0) - 2\gamma_{4,0} I_{ICU,1}(t,a,v_5,1,0) + 2\psi I_{ICU,1}(t,a,v_4,1,0)$$

$$\frac{dI_{ICU,0}(t,a,v_5,0,1)}{dt} = (1-\delta(ICU))(1-\mu(a))\phi_2(a)2\gamma_2 I_{CASE,1}(t,v_5,a) - 2\gamma_{4,1} I_{ICU,0}(t,a,v_5,0,1) + 2\psi I_{ICU,0}(t,a,v_4,0,1)$$

$$\frac{dI_{ICU,1}(t,a,v_5,0,1)}{dt} = 2\gamma_{4,1} I_{ICU,0}(t,a,v_5,0,1) - 2\gamma_{4,1} I_{ICU,1}(t,a,v_5,0,1) + 2\psi I_{ICU,1}(t,a,v_4,0,1)$$

$$\frac{dI_{ICU,0}(t,a,v_5,1,1)}{dt} = \delta(ICU)(1-\mu(a))\phi_2(a)2\gamma_2 I_{CASE,1}(t,a,v_5) - 2\gamma_{4,1}I_{ICU,0}(t,a,v_5,1,1) + 2\psi I_{ICU,0}(t,a,v_4,1,1)$$

$$\frac{dI_{ICU,1}(t,a,v_5,1,1)}{dt} = 2\gamma_{4,1}I_{ICU,0}(t,a,v_5,1,1) - 2\gamma_{4,1}I_{ICU,1}(t,a,v_5,1,1) + 2\psi I_{ICU,1}(t,a,v_4,1,1)$$

$$\frac{dI_{REC,0}(t,a,v_5)}{dt} = 2\gamma_{4,1}I_{ICU,1}(t,a,v_5,0,1) + 2\gamma_{4,1}I_{ICU,1}(t,a,v_5,1,1) - 2\gamma_5 I_{REC,0}(t,a,v_5) + 2\psi I_{REC,0}(t,a,v_4)$$

$$\frac{dI_{REC,1}(t,a,v_5)}{dt} = 2\gamma_5 I_{REC,0}(t,a,v_5) - 2\gamma_5 I_{REC,1}(t,a,v_5) + 2\psi I_{REC,1}(t,a,v_4)$$

$$\frac{dR_1(t,a,v_5)}{dt} = \gamma_1 I_{MILD}(t,a,v_5) + 2\gamma_{3,1}I_{HOSPITAL,1}(t,a,v_5,0,1) + 2\gamma_{3,1}I_{HOSPITAL,1}(t,a,v_5,1,1) + 2\gamma_5 I_{REC,1}(t,a,v_5) + 2\gamma_{4,1}I_{ICU,1}(t,a,v_5,0,1) + 2\gamma_{4,1}I_{ICU,1}(t,a,v_5,1,1) - 2\rho R_1(t,a,v_5) + 2\psi R_1(t,a,v_3)$$

$$\frac{dR_2(t,a,v_5)}{dt} = 2\rho R_1(t,a,v_5) - 2\rho R_2(t,a,v_5) + 2\psi R_2(t,a,v_3)$$

$$\frac{dD(t,a,v_5)}{dt} = 2\gamma_{3,0}I_{HOSPITAL,1}(t,a,v_5,0,0) + 2\gamma_{3,0}I_{HOSPITAL,1}(t,a,v_5,1,0) + 2\gamma_{4,0}I_{ICU,1}(t,a,v_5,0,0) + 2\gamma_{4,0}I_{ICU,1}(t,a,v_5,1,0)$$

Table S1. Estimated biweekly time–varying reproduction number (R_t), starting from March 1, 2020, to December 31, 2022.

**Time since March 1, 2020 (days)**	**Median R_t (95% CI: Lower–Upper)**
0	2.50 (2.49–2.51)
14	1.85 (1.84–1.86)
28	1.85 (1.84–1.86)
42	1.75 (1.74–1.76)
56	1.15 (1.14–1.16)
70	0.90 (0.89–0.91)
84	0.90 (0.89–0.91)
98	0.95 (0.94–0.96)
112	0.95 (0.94–0.96)
126	0.85 (0.84–0.86)
140	0.85 (0.84–0.86)
154	0.85 (0.84–0.86)
168	0.85 (0.84–0.86)

182	0.75 (0.74–0.76)
196	0.75 (0.74–0.76)
210	0.75 (0.74–0.76)
224	0.75 (0.74–0.76)
238	0.75 (0.74–0.76)
252	0.80 (0.79–0.81)
266	0.80 (0.79–0.81)
280	1.50 (1.49–1.51)
294	1.55 (1.54–1.56)
308	1.15 (1.14–1.16)
322	1.29 (1.28–1.30)
336	1.33 (1.32–1.34)
350	1.23 (1.22–1.24)
364	1.21 (1.20–1.22)
378	1.21 (1.20–1.22)
392	1.31 (1.30–1.32)
406	1.29 (1.28–1.30)
420	1.40 (1.39–1.41)
434	1.35 (1.34–1.36)
448	1.30 (1.29–1.31)
462	1.38 (1.37–1.39)
476	1.40 (1.39–1.41)
490	1.40 (1.39–1.41)
504	1.30 (1.29–1.31)
518	1.30 (1.29–1.31)
532	0.98 (0.97–0.99)

546	1.10 (1.09–1.11)
560	1.55 (1.54–1.56)
574	1.70 (1.69–1.71)
588	2.10 (2.09–2.11)
602	2.00 (1.99–2.01)
616	1.98 (1.97–1.99)
630	2.00 (1.99–2.01)
644	2.00 (1.99–2.01)
658	2.10 (2.09–2.11)
672	2.25 (2.24–2.26)
686	2.40 (2.39–2.41)
700	2.40 (2.39–2.41)
714	2.50 (2.49–2.51)
728	2.40 (2.39–2.41)
742	2.20 (2.19–2.21)
756	2.10 (2.09–2.11)
770	1.20 (1.19–1.21)
784	1.35 (1.34–1.36)
798	1.40 (1.39–1.14)
812	1.50 (1.49–1.51)
826	1.70 (1.69–1.71)
840	1.90 (1.89–1.91)
854	2.30 (2.29–2.31)
868	1.70 (1.69–1.71)
882	1.26 (1.25–1.27)
896	1.37 (1.36–1.38)

910	1.35 (1.34–1.36)
924	1.20 (1.19–1.21)
938	1.10 (1.09–1.11)
952	1.40 (1.39–1.41)
966	1.80 (1.79–1.81)
980	1.95 (1.94–1.96)
994	1.50 (1.49–1.51)
1008	0.90 (0.89–0.91)
1022	0.85 (0.84–0.86)
1036	0.50 (0.49–0.51)

Table S2. Model parameters, values, and definitions used in the COVID-19 transmission model.

Parameters	Values	Definition
Time-varying reproduction number (R_t)	Table S1	A number of secondary infections generated by an infectious individual at a given time.
Transmission rate	Calculated from R_t	
Mean latent period	4.6 days	Average time from exposure to the virus until the onset of infectiousness.

Mean duration of mild infection	2.1 days	Average time a patient with mild symptoms remains infectious. Mild infection is defined as cases with not very strong or severe symptoms that do not require hospitalization.
Mean duration of severe infection prior to hospitalization	4.5 days	Average time from the onset of severe symptoms to hospitalization. Severe infection is characterized by cases with severe symptoms requiring medical intervention and ICU care.
Mean duration of hospitalization for non-severe cases if survive	9.0 days	Average time of hospital stay for non-severe patients who recover. Non-severe cases refer to those who are hospitalized but do not require critical care (ICU with ventilator care) for life support.
Mean duration of hospitalization for non-severe cases if die	9.0 days	Average time of hospital stay for non-severe patients who do not survive.
Mean duration in ICU if survive	14.8 days	Average duration a patient spends in ICU if they recover.
Mean duration in ICU if die	11.1 days	Average duration a patient spends in ICU if they do not survive.
Mean duration in recovery after ICU	3.0 days	Average duration a patient spends recovering post-ICU before hospital discharge.

Mean vaccine efficacy against infection	60%	
Mean vaccine efficacy against disease	70%	
Mean duration of naturally acquired immunity	365 days	Average duration of recovered individuals maintain immunity against COVID-19 reinfection.
Vaccine duration of protection	446 days	Average duration of immunity provided by COVID-19 vaccination.
Probability of death if require critical care but do not receive it	90.5% (range 85%–95%)	
Probability of death if require hospitalization but no hospital beds are available	60% (range 50–70%)	
Number of hospital beds in Thailand	158,326	The total number of available hospital beds across Thailand, as accounted for in the model.
Number of ICU beds with ventilators in Thailand	13,184	Total number of ventilator-equipped ICU beds across Thailand provided for severe COVID-19 cases requiring critical care for life support, including ventilator support for breathing.

Reference

1. Hogan, A.B.; Winskill, P.; Watson, O.J.; Walker, P.G.; Whittaker, C.; Baguelin, M.; Brazeau, N.F.; Charles, G.D.; Gaythorpe, K.A.; Hamlet, A. Within-country age-based prioritisation, global allocation, and public health impact of a vaccine against SARS-CoV-2: A mathematical modelling analysis. *Vaccine* **2021**, *39*, 2995-3006, doi:10.1016/j.vaccine.2021.04.002.
